# Assessing the readiness and feasibility to implement a model of care for spine disorders and related disability in Cross Lake, an Indigenous community in northern Manitoba, Canada: a research protocol

**DOI:** 10.1186/s12998-025-00576-1

**Published:** 2025-03-13

**Authors:** André Bussières, Steven Passmore, Deborah Kopansky-Giles, Patricia Tavares, Jennifer Ward, Jacqueline Ladwig, Cheryl Glazebrook, Silvano Mior, Melissa Atkinson-Graham, Jean Moss, Nicole Robak, Elena Broeckelmann, David A. Monias, Donnie Z. Mckay, Helga Hamilton, Muriel Scott, Randall Smolinski, Eric L. Hurwitz, Anthony D. Woolf, Michael Johnson, Melinda J. Fowler-Woods, Scott Haldeman

**Affiliations:** 1 Département chiropratique, Université du Québec à Trois-Rivières, Trois-Rivières, QC Canada; 2 School of Physical and Occupational Therapy, Faculty of Medicine and Health Sciences, McGill University, Montreal, QC Canada; 3 World Spine Care Canada, Toronto, ON Canada; 4 Faculty of Kinesiology and Recreation Management, University of Manitoba, Winnipeg, MB Canada; 5 Graduate Education and Research, Canadian Memorial Chiropractic College, Toronto, ON Canada; 6 Ontario Tech University, Oshawa, ON Canada; 7 Department of Family and Community Medicine, University of Toronto, Toronto, ON Canada; 8 Pimicikamak Okimawin, Cross Lake Band of Indians, Cross Lake, MB Canada; 9 Cross Lake Health Services, Cross Lake, MB Canada; 10 Cross Lake Nursing Station, Cross Lake, MB Canada; 11 Department of Public Health Sciences, University of Hawai’i at Mānoa, Honolulu, HI USA; 12 World Spine Care, Tustin, CA USA; 13 Bone and Joint Research Group Knowledge Spa, Royal Cornwall Hospital, Treliske, Truro, UK; 14 Department of Surgery, Rady Faculty of Health Sciences, University of Manitoba, Winnipeg, MB Canada; 15 Winnipeg Spine Program, Winnipeg, MB Canada; 16 University of California, Irvine, CA USA

**Keywords:** Participatory mixed-methods, Spine care, Value-based healthcare, Implementation science, Medically underserved area, Vulnerable population, Protocol, Chiropractic, Indigenous

## Abstract

**Background:**

Since the 1990s, spine disorders have remained the leading cause of global disability, disproportionately affecting economically marginalized individuals, rural populations, women, and older people. Back pain related disability is projected to increase the most in remote regions where lifestyle and work are increasingly sedentary, yet resources and access to comprehensive healthcare is generally limited. To help tackle this worldwide health problem, World Spine Care Canada, and the Global Spine Care Initiative (GSCI) launched a four-phase project aiming to address the profound gap between evidence-based spine care and routine care delivered to people with spine symptoms or concerns in communities that are medically underserved. Phase 1 conclusions and recommendations led to the development of a model of care that included a triaging system and spine care pathways that could be implemented and scaled in underserved communities around the world.

**Methods:**

The current research protocol describes a site-specific customization and pre-implementation study (Phase 2), as well as a feasibility study (Phase 3) to be conducted in Cross Lake, an Indigenous community in northern Manitoba, Canada. *Design:* Observational pre-post design using a participatory mixed-methods approach. Relationship building with the community established through regular site visits will enable pre- and post-implementation data collection about the model of spine care and provisionally selected implementation strategies using a community health survey, chart reviews, qualitative interviews, and adoption surveys with key partners at the meso (community leaders) and micro (clinicians, patients, community residents) levels. Recruitment started in March 2023 and will end in March 2026. Surveys will be analyzed descriptively and interviews thematically. Findings will inform co-tailoring of implementation support strategies with project partners prior to evaluating the feasibility of the new spine care program.

**Discussion:**

Knowledge generated from this study will provide essential guidance for scaling up, sustainability and impact (Phase 4) in other northern Canada regions and sites around the globe. It is hoped that implementing the GSCI model of care in Cross Lake will help to reduce the burden of spine problems and related healthcare costs for the local community, and serve as a scalable model for programs in other settings.

**Supplementary Information:**

The online version contains supplementary material available at 10.1186/s12998-025-00576-1.

## Background

Musculoskeletal disorders affect over 1.71 billion people worldwide, [1] and are the leading contributor to disability. Disability is amplified in remote communities and low-middle income countries (LMICs) where access to care and health resources are limited. [2, 3] Among musculoskeletal disorders, spine pain remains the leading cause of global disability since 1990, [4–6] and is one of the most common complaints seen by primary care clinicians [7]. Moreover, spine pain accounts for nearly 50% of all opioid prescriptions. [7, 8] Spine disorders disproportionately affect economically marginalized individuals, rural populations, women, and older people. [4, 5] Because of population growth and ageing, the number of people living with spine pain and associated disability is rapidly increasing with projections of 843 million people living with low back pain [4] and 269 million people having neck pain by 2050. [9] Hence, spine pain is expected to place an ever-increasing demand on health systems that are already challenged to support appropriate and timely treatment for spine pain and disability [2, 10, 11].

Despite musculoskeletal disorders posing significant burdens to individuals, communities and economies, they have received minimal attention from global and national policy makers. [12, 13] To tackle the world-wide problem of spine disorders, World Spine Care (WSC), a multinational, not-for-profit, charitable organization, has been delivering spine services for the past 15 years in Botswana, the Dominican Republic, India, and Ghana in collaboration with the local community and with governmental support. [14] The WSC program has been recognized by the World Health Organization (WHO) Integrated, People-Centred Health Services (IPCHS) program as a global promising practice [15].

In 2018, the WSC’s Global Spine Care Initiative (GSCI) published a series of papers describing a new model of spine care (MoC) with the flexibility to be implemented in any region of the world. [16–29] The MoC outlines the most up to date evidence-based spine care and services for a person or a population group as they progress through the stages of a condition, injury or event to ensure “*that people get access to care and get the right care, at the right time*, *by the right team, and in the right place*" and helps guide policy makers to transform care in order to address a specific health concern [29, 30].

The activities and initiatives of WSC and the GSCI are consistent with the United Nations’ Sustainable Development Goals (SDG) as they help to mitigate the impact of spine conditions on peoples’ health (SDG Target 3.4), and promote healthy lives and well-being for all (Goal 3) [31] However, the MoC needs to be rigorously tested with a focus on implementation, sustainability, scalability, and impact on individuals, their families, and healthcare systems, particularly in underserved communities. Implementation requires addressing important contextual factors to accessing healthcare interventions, such as clinician and patient attitudes, traditional beliefs, socio-cultural norms and behavior. System level barriers include lack of support or interest from government ministries, human resource shortages, high patient out-of-pocket costs, lack of conveniently located facilities, gender discrimination, or cultural values and preferences of communities [32–36].

Both in Canada and internationally, colonization has been recognized as a having a fundamental impact on the health of Indigenous peoples. [37, 38] Examples of discrimination in health care against Indigenous peoples in Canada are well documented, and often involve the mismanagement of pain, [39–41] with for instance, opioid-related overdose events more likely to occur among First Nations people in Western Canada than their non–First Nations counterparts. [42] Comprehensive care for Indigenous peoples includes access to family, community, traditions and ceremonies, all of which are central to healing. Yet many Indigenous persons, especially those who live in rural or remote communities, are often required to travel long distances to receive services, leading to removal from their community and/or family support system, and high costs. These issues may also be compounded by language barriers and difficulty in accessing culturally safe and meaningful health care services. [43] Promising and emerging responses aligning with the Truth and Reconciliation Commission of Canada, [44] include Indigenous directed health and health related services, efforts to increase the number of Indigenous health care providers, cultural safety training and trauma-informed care, and interventions addressing implicit (unconscious, pro-settler) bias of care providers to reduce health inequities and provide the best care [45].

We acknowledge the inherent differences between Western methods and Indigenous ways of knowing and the risks in trying to integrate these approaches (e.g., generalizing Indigenous traditions by taking them out of context; denying cultural differences in order to find commonality; assimilating Indigenous knowledge in a way that it becomes invisible). [46] To address power imbalances and philosophical differences, the team will seek to understand, with humility and respect, Indigenous knowledges and ways of knowing. Through discussion, we will select Indigenous-Western knowledge linking frameworks (principles and methods), [47] such as *Etuaptmumk* (Two-Eyed Seeing), [48] considering specific context (history, place, distinct character, and beliefs of the Indigenous community), and seek to adopt seven principles found in Indigenous and Western science (Relationality, Reciprocity, Reflexivity, Respect, Reverence, Responsivity, and Responsibility), helping political, academic and other actors fulfill their obligations to both truth and reconciliation and gender-based analysis plus policies and practices [47].

Following established principles to guide ethical research within Canadian Indigenous communities, [49] team members completed recommended training (Tri-Council Policy Statement (TCPS 2), First Nations Principles of OCAP, Personal Health Information Act) prior to obtaining ethics approval from the University of Manitoba’s Research Ethics Board for each study component. Applying the aforementioned principles and teachings, we conceptualized a four-phase program of care to address the increasing burden of spine pain in underserved communities. In this protocol, we focus on Phases 2 and 3 that will be conducted over a 3-year period and reported in accordance with the requirements of the Standards for Reporting Implementation Studies (StaRI) Statement [50].

### Aims and objectives

**Phase 1**—involved creating an evidence-based Model of Spine Care (MoC), completed in 2018 by the GSCI, which included a triaging system and care pathways to be implemented in underserved communities. [16–29]. **Phase 2**—focuses on site-specific customization and pre-implementation, tailoring the MoC to meet local needs. It includes three studies: two already completed, [51, 52] which provided foundational insights, and a third outlined in this protocol. **Phase 3**––feasibility study describes the implementation of the MoC in Cross Lake, Manitoba. Knowledge gained will inform **Phase 4**, which aims to scale up, sustain, and assess the impact of the MoC in underserved communities across northern Canada and globally.

The objectives of each study phase are:

### Phase 2. Site-specific customization and pre-implementation

1. Confirm the nature of, and extent to which, spinal disorders impact individuals within the underserved community.
2. Measure the perceived value of, and intention to adopt, the MoC triaging system and tailored care approach. Engage with community partners to identify factors that may impact MoC implementation.
3. Estimate the extent of: (I) community partners support and engagement throughout the pre-implementation phase; (II) local clinicians and caregivers adoption and application of the MoC as intended; and (III) people with spine symptoms would utilize the MoC.

### Phase 3. Feasibility to implement the GSCI MoC

4. Identify, estimate, and understand the extent to which: (i) pain and related disability outcomes are important to people with spine symptoms or concerns; and (ii) if and how the MoC can be integrated into new or existing community-based programs.
5. Estimate key parameters to inform a future Phase 4 (upscaling) project.

## Methods/design

**Fig. 1:**
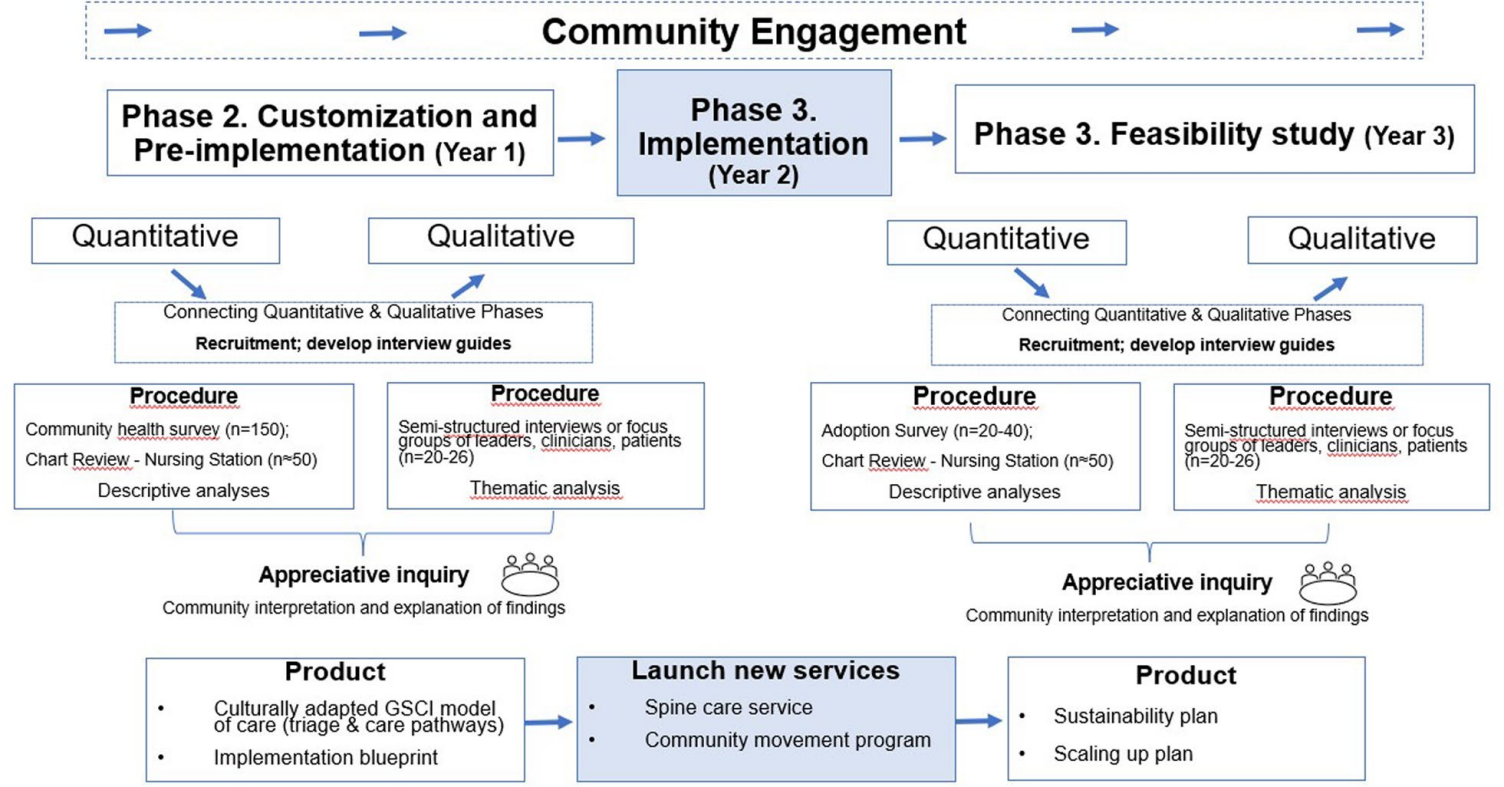
Mixed-method sequential exploratory design flow chart

Observational pre-post design, using participatory, sequential mixed-methods approaches. [53, 54] Mixed-methods research uses quantitative and qualitative research integration to develop contextual understanding of complex multi-level systems. [53] Participatory research involves the co-production of knowledge that is relevant to policy and practice, with an explicit focus on end users’ concerns, participation, and outcomes to enable practice change by empowering those most likely to use the new knowledge. [55, 56] The research team will actively engage local partners and Knowledge Keepers throughout the study at the meso- (community leaders, health administrators), and the micro-levels (local clinicians, people with spine problems, community residents), and collect quantitative followed by qualitative information (Fig. 1).

### Setting

Indigenous populations in Manitoba, especially those in the northern communities, bear an excessive burden of injury, and acute and chronic diseases compared with the Canadian population as a whole. These groups have limited access to health care, and serious illnesses require patients to fly to southern Manitoba for care. [43, 57] A GSCI team member (JW), an Indigenous chiropractor with over 20 years’ experience delivering spine care near The Pas on Opaskwayak Cree Nation, in northern Manitoba, was instrumental in engaging community leaders of Cross Lake Band/Pimicikamak Cree Nation (https://crosslakeband.ca/), an Indigenous community committed to increasing healthcare services. Since its first site team visit in April 2022, and in accordance with the University of Manitoba Framework for Research Engagement with First Nation, Metis, and Inuit Peoples, members of the GSCI have developed a strong partnership with Cross Lake community where the GSCI MoC will be implemented. Cross Lake is located 786 km north of Winnipeg, the capital of Manitoba. Cross Lake has an on-reserve population of 6,734 and an off-reserve population of 2715. [58] Inhabitants include First Nations peoples, Métis, and people of non-Indigenous origin. The majority of the population maintain treaty status and the Indigenous language most commonly spoken locally is Cree. Those in need of spine care must travel to nearby cities. Both The Pas (a 401 km drive) and Thompson (a 255 km drive) have hospitals with access to telehealth for specialized services available in Winnipeg, including orthopedic spine surgeons. More serious illnesses and spine care needs require residents to fly to Winnipeg (520 km).

### Participants

*Community health survey, new clinical service and community movement program:* All adults 18 years and older with spine symptoms (pain, disability) or concerns (e.g., prior problems with their neck or back), with or without radiating pain, residing in Cross Lake, Manitoba, will be eligible for inclusion. Translation will be used with adults who prefer to communicate in Cree.

*Chart reviews:* Clinical records of consenting consecutive adult patients presenting with a chief complaint of musculoskeletal pain at the Cross Lake Nursing Station run by Health Canada over a 12-month period will be accessed and de-identified.

*Adoption survey, qualitative interviews or focus groups:* Partners likely to influence implementation of the MoC including the Chief and community Band council members, community Elders and knowledge keepers, as well as Directors of Cross Lake Health Services, representatives of the local government, and all licensed local care providers (e.g., medical doctors, nurses, allied health care providers) and local community health workers (CHWs), exercise therapists, massage therapists, and traditional healers will be eligible for inclusion.

### Study sample

*Community health survey*: 50 homes in the community were randomly selected from the treaty (n = 1931 houses) and non-treaty (n = 92 houses) land, using a household list available from the community’s urban planning. A trained local RA (MS) orally administered the CHS at the study onset to up to three adults living in these households (total of 150 surveys). A sample size estimate determined that 96 respondents is needed for a population of 10,000 at 90CI% (margin of error = 10%) for this exploratory study. To account for participant attrition and the effect of missing data, we recruited 30% more participants in this study.

*Chart reviews*: For this retrospective exploratory chart review, up to 100 consecutive patient charts will be extracted by team members (JW, PT), including 50 charts pre- and 50 charts post-implementation (or until data saturation) to provide a portrait of musculoskeletal care delivered. A sample size of 7–10 cases (charts) per variable (n = 9) is deemed acceptable to obtain results that are likely to be both true and clinically useful [59].

*Adoption surveys*: Between 4 and 8 participants per partner group (decision-makers/local leaders; local clinicians/CHWs, people with spine symptoms, community residents) will complete 3 short implementation surveys post-implementation, either paper-based or orally administered by RAs.

*MoC Fidelity checklists:* GSCI primary spine care clinicians will complete the fidelity checklists while observing 4–6-consecutive clinical encounters between the local clinicians and CHWs (n = 6–8 per setting) and people seeking spine care 3 and 6 months after online and in-person training.

*Qualitative interviews and focus groups*: Semi-structured interviews or focus groups conducted pre- and post-implementation by co-authors (AB, JL) in English or in the local language by a local RA (MS):

i) *Semi-structured interviews:* Using maximum variability principles, a purposive sample of 10–13 individuals will be drawn among community leaders (n = 10), respondents of the chart review (n = 2–3) and community health survey (n = 4–5) to seek respondents across a spectrum (spread of age, gender, occupation, pain duration, disability level) to ensure that all viewpoints would be adequately represented;
ii) *Focus group or semi-structured interviews* conducted with 6–8 local clinicians and 1–2 CHWs representing a wide range in years in practice and health disciplines.

*Community movement program:* During an ‘open house/information session’ hosted over 2 days by co-authors (JL, CG, MS), community residents were invited to participate in a movement program demonstration. Up to 20 interested adults will be invited to undertake a virtual dance-based movement program involving two 60-min sessions per week for 12–15 weeks (total of 24–30 sessions) in a group setting in Cross Lake.

*Clinical service:* Consecutive spine pain patients seeking chiropractic care at the Nursing Station will be invited to complete a consent form and patient reported outcome measures before and after care. There is no sample size for the clinical service as there are no hypothesis.

### Recruitment

Participants recruitment to take place between March 2023 and March 2026. All participants will receive $25 gift cards to the local grocery store in compensation for their time.

#### Phase 2. Customization and pre-implementation

Community residents were invited via the Cross Lake private Facebook page and radio announcements to participate in an orally administered community health survey in English or in Cree. Individuals with a history of spine pain, who sought care, and who agreed to be contacted, will be asked to give permission for a research team member to access their chart at the Nursing Station- we will obtain signed informed consent from before accessing any patient charts. Nursing Station clinicians and community leaders were invited to participate in qualitative interviews or focus groups.

#### Phase 3. Feasibility study

Community residents will be invited to participate the community movement program via social media and the local radio. Consecutive patients who are referred or self-referred to the clinical service will be invited to sign a consent form and complete patient reported outcome measures. At the end of the study, Nursing Station clinicians and community leaders will again be invited to complete the adoption survey and take part in end of study qualitative interviews.

### Procedures, data collection, evaluation

Table 1 outlines the project phases, methods for data collection and designated timelines. Study instruments are available upon request.

**Table 1:** Description of project phases, timing, data collection

Project phases	Duration	Activities
Phase 2. Customization and pre-implementation	12 months	Build local implementation team Periodic community engagement site visits Secure REB approvals, Memorandum of Understanding, and data sharing agreement Co-create, refine, culturally adapt, and prepare all study material, select patient health outcome measures Design database linkages Train research assistants Recruit study participants for the chart review, Community Health Survey, and qualitative interviews; analyze & interpret data Prepare community movement program; select primary spine care clinicians Online and in-person educational training of local clinicians and community health workers on GSCI triaging and spine care pathways Fidelity & monitoring log (FRAME adaptation framework)
Phase 3. Feasibility Implementation	10 months	Further customize MoC tools and implementation support strategies Implement protocols Launch community education and movement program Launch new clinical service for people with spine symptoms or concerns Collect implementation, service and patient outcomes Implementation adoption survey (AAF tool) MoC Fidelity checklist, feedback, local team meetings Fidelity & monitoring log (FRAME adaptation framework)
Post-implementation	12 months	Post-implementation visit Second chart review and qualitative interviews Fidelity & monitoring log (FRAME adaptation framework)
Data analysis and interpretation (pre- and post-implementation)	6 months	Conduct quantitative and qualitative analyses (pre- and post-implementation) Meet with key stakeholders to help with data interpretation (both pre- and post-implementation data) Fidelity & monitoring log (FRAME adaptation framework)
Dissemination plan Prospect for sustainability and GSCI Initiative Phase 4	2 months	Meet with health authorities (district, provincial, Federal level) to discuss sustainability of services within the community, and scaling up in other communities

AAF, Acceptability, Appropriateness, Feasibility; FRAME, Framework for Reporting Adaptations and Modifications-Enhanced; GSCI, Global Spine Care Initiative; MoC, Model of Care; REB, Research Review Board; Stepwise approach to (1) prepare implementation, assessing the current status of spine care in Cross Lake; identifying potential barriers and facilitators to the uptake of the MoC, and co-designing implementable solutions, and (2) evaluate the feasibility to implement a new spine care clinical service and community movement program supported by tailored strategies

### Phase 2. Customization and pre-implementation (Year 1)

#### Quantitative data

At the study onset, we will conduct a community health survey and a retrospective chart review to confirm the nature of and extent to which spinal disorders impact individuals within selected underserved communities.

*Community health survey:* The survey questionnaire contained 154 questions derived from the 2020 Canadian Community Health Survey [60] and the Global Burden of Disease [61], covering socio-demographics, general health, spine pain and related disability, chronic comorbid conditions, self-care, spine care received, and satisfaction with care.

*Chart review:* De-identified data on consecutive charts from adult community members seeking musculoskeletal care within the preceding year at Cross Lake Nursing Station will be reviewed to access patient demographics and spine-related health care utilization (pain location, duration, and intensity, spine-related disability, prescribed imaging and medication, and patient referrals).

#### Qualitative data

*Semi-structured individual in-person or online interviews* or focus groups of community leaders, local clinicians and CHWs were conducted to better understand the intention and readiness to adopt the MoC, and explore individual, organizational, and contextual factors shaping the uptake of MoC within the community. [62] The interview guides was informed by the Theoretical Domain Framework (TDF) [63], offering an ecological lens in which to consider multi-level influences on behavior change. [64] The TDF has been widely used across health disciplines, health conditions and settings. The framework guided the data collection, coding, analysis, and reporting of findings to gain a comprehensive understanding of relevant modifiable determinants, to facilitate the design of implementation strategies that will address them [65].

### Phase 3. Feasibility study (Year 2–3)

#### Implementation (Year 2)

Phase 3 will begin after baseline data collection, the MoC and related implementation strategies have been refined, culturally adapted, and prepared for initiating the implementation. Local clinicians will have been trained to apply the GSCI triage and care pathways on patients with spine symptoms or concerns (Additional file 1, Appendix 4, Table 1–3). In parallel, we will prepare and launch the new spine care service and community movement program (Additional file 1, Appendix 5).

#### Post-implementation (Year 3)

##### Quantitative data

We will administer adoption survey questionnaires to meso- and micro-level partners 6 months after initiating implementation of the MoC, inquiring about the perceived acceptability, appropriateness, and feasibility to implement the MoC and related implementation support strategies. These three brief measures will be collected after implementation of the new clinical service and community movement program as users need to first experience the MoC (triage and care pathways) and implementation strategies (e.g., educational training modules, self-care tools) and have initiated the use of the MoC prior to completing these questionnaires.

A second *chart review* will be conducted 9–12 months post-implementation to estimate and understand the extent to which: (i) local clinicians and caregivers accepted, adopted, and applied the GSCI MoC as intended; and (ii) people with musculoskeletal symptoms have accepted recommended care.

*Service and clinical outcomes:* At the end of each patient care episode for a complaint of spine symptoms, patient charts will be reviewed to determine the care delivered (i.e., process through which patients with spine condition are diagnosed, treated, referred or managed over time) along with the patient’s self-reported outcome measures. Validated patient reported health outcome questionnaires (pain, function, disability, quality of life) will be administered before and after care over a 2-month period. The results from the patient health outcome measures will be used to estimate key parameters such as effect sizes to inform the selection of a primary outcome and to calculate the sample size for a future study.

##### Qualitative data

We will conduct a second series of in-person TDF-guided interviews and focus groups [62] among the same partners 9–12 months after initiating implementation, to understand if and how the MoC was implemented, gather information on local clinicians’ and CHWs’ intentions to continue use of the MoC, community leaders’ willingness to maintain the new clinical service and community movement program, and community leaders’ priority toward helping scale up this project in other communities.

### Data sources

The aforementioned objectives will be addressed using the following data sources (Table 2).

**Table 2:** Data sources

Study objectives	Level	Data collected	Tools	Outcome	How it is done
*Phase 2. Customization and pre-implementation*					
Objectives 1. Confirm the nature of and extent to which spinal disorders impact individuals in Cross Lake, northern Manitoba, Canada	Micro level	Quantitative Quantitative	Community health survey Retrospective chart review	Perceived impact	Trained RA
Objectives 2. Measure the value of, and intention of partners to adopt the GSCI MoC, and identify barriers and facilitators impacting the implementation of this model among partners	Meso- and micro level	Qualitative	Semi-structured interviews or Focus groups	Perceived TDF barriers and enablers	Project PI, trained RA
Objectives 3. Estimate and understand the extent to which (i) partners support and engage throughout the study, and are empowered to drive the identification and refinement of implementation strategies for their own barriers, (ii) local clinicians and caregivers accept, adopt, and apply the GSCI MoC as intended, and (iii) people with spine symptoms accept recommended care	(i) Meso- and (ii, iii) micro level	Quantitative	Administrative data (support letters, signed MOU, meeting attendance, THET-Partnership-Health-Check-Tool) Education training attendance	Reach Adherence/Fidelity	Local implementation team Online & in-person educational modules
*Phase 3. Feasibility study*					
Objectives 4. Identify, estimate, and understand the extent to which: i) pain and related disability outcomes are important to people with spine symptoms or concerns; and ii) if and how the MoC can be integrated into new or existing community-based programs	(i, iii) Micro-level (ii) Meso- and micro level	Qualitative Quantitative	Semi-structured interviews Adoption surveys; Retrospective chart review	Perceived TDF barriers and enablers Adoption (AAF measures) Implementation (Fidelity, Perceived cost)	PI, trained RA Online, paper-based, and orally administered
Objectives 5. Estimate key parameters such as effect sizes to inform the selection of a primary outcome and to calculate the sample size for a future Phase 4 implementation study	All levels	Quantitative	Surveys Clinical outcomes	Fidelity and context monitoring log and a fidelity checklist Pain, function, disability, quality of life	Co-PIs, statistician Local clinicians, RA

AAF, Acceptability, Appropriateness, Feasibility; GSCI, Global Spine Care Initiative; MoC, Model of Care; MOU, Memorendum of understanding; PI, Principal Investigator; RA, Research assistant; TDF, Theoretical Domain Framework

## Implementation and secondary outcomes

The study outcomes were selected to reflect the hypothesized mechanism of effect of the proposed implementation support strategies of the MoC, while considering the need to minimize respondent burden and maintain participant confidentiality. The measures have established psychometric properties, and can be compared when the MoC is implemented in other settings [66–71].

Drawing on the RE-AIM evaluation framework, study outcomes will focus on reach, adoption, implementation, and maintenance. [72–74] The Proctor et al. [75] and Lewis et al. [76] taxonomies will serve to further characterize selected primary process outcomes, downstream service, and clinical outcomes measures. Further, we will use appreciative inquiry [77] involving a four phase cycle (i.e., Discovery ‘*valuing the best of what is’*, Dream *‘envisioning what might be’*, Design *‘dialoguing what should be’*, and Delivery/Destiny *‘innovating what will be’*) [78, 79] to reflect on the extent to which the proposed GSCI MoC and services align with key concepts of the Indigenous Healthcare Quality Framework. [79] This framework represents the person-centered perspectives and the requirements of healthcare systems and provider factors that are required for the achievement and sustainability of health care for Indigenous people that is high quality, culturally safe and free of racism. It also considers the continuous cycles experienced throughout the lives of Indigenous people, and the vital connection to the land held by First Nations, Inuit, and Metis peoples.

### Implementation outcomes

*Reach* can be defined as “…the integration of a practice within a service setting and its subsystems”. [75] We will adapt the THET-Partnership-Health-Check-Tool to evaluate stakeholders engagement [80] using quantitative methods (e.g. administrative data such as support letters, formal memoranda of understanding, meeting attendance, and partners adoption survey). [81] *Adoption* relating to the readiness for implementation [82] will be gathered using validated 4-item measures of acceptability of intervention measure (AIM), intervention appropriateness measure (IAM), and feasibility of intervention measure (FIM). [83, 84] *Acceptability* is the perception among stakeholders that the intervention is agreeable, palatable, and satisfactory. *Appropriateness* is the perceived fit, relevance, or compatibility of the innovation for a given setting, provider, or consumer. *Feasibility* is the extent to which the intervention can be successfully carried out within the given setting. [85] The implementation measures are scored with a 5-point Likert scale (1 = strongly disagree to 5 = strongly agree), with higher average scores indicating greater readiness for implementation. These measures have demonstrated strong psychometric properties, and readability is at the 5th grade level. [83] *Implementation (adaptability, fidelity)* is the consistency at which the different parts of the GSCI triage and care pathways are implemented across settings, clinicians, and patients, and at what cost, and how was the program adapted. [74] *Adaptability* can be defined as “the degree to which an intervention can be adapted, tailored, refined, or reinvented to meet local needs”, while *Fidelity* “the degree to which an intervention was implemented as intended”. [75] An Adaptation framework will capture adaptations made during the study. An Implementation Status Report will collect clinical and implementation activities. *Cost* are the resources (personnel, material) utilized by the strategies and their costs, [86] including the delivery of the new services, staff/clinician training, patients’ external referrals for advanced imaging and medical specialist consultation, and travels.

### Secondary outcomes

*Service outcomes* refers to local clinicians delivering care during clinical encounters, people with spine symptoms applying advice on self-care (e.g., home exercise), and community members attending activities (e.g., community movement program). Service outcomes may be determined by an observer with some professional training or self-reported using the MoC fidelity checklist. *Clinical outcomes* are considered secondary outcomes as the aim is to determine whether questionnaires can be routinely collected as planned. When a patient attends for consultation, socio-demographic, baseline and follow-up measures will be obtained using validated questionnaires: (a) Numeric rating scale (NRS) to assess pain; (b) WHODAS 2.0 (WHO Disability Assessment Schedule 2.0) to measure ability; (c) Patient Specific Functional Scale for function; and (d) EuroQol (EQ-5D 3L) for health-related quality of life. We recognize however the need to discuss with our partners the selection of culturally adapted outcome measures. [87] For instance. Indigenous perspectives of pain are often more holistic, encompassing mental, spiritual, emotional, and physical hurt [88].

## Analysis

Using appreciative inquiry, [77] we will seek input from our partners in the interpretation of the findings and dissemination and implementation of the research results. Where available, analyses will consider sex, gender and age-related differences and patterns in the data. We will complete yearly implementation status reports, including a model of care matrix, partner’s analysis table, and implementation strategy plan.

### Quantitative data analysis

Statistical analyses will be conducted using SAS Analytics Software (SAS Institute). Data from the Community health survey (CHS), chart reviews (CR), and implementation measures will be analyzed descriptively. Frequency distributions and proportions will be generated for categorical variables, and means, standard deviations, and medians with interquartile ranges will be computed for continuous variables.

For the CHS, we analyzed three self-reported measures of in-community spine symptoms in the 12 months prior to the survey:

i) Whether the respondent reduced their participation or level of activity as a result of spine symptoms and related co-morbidities,
ii) Whether the respondent consulted someone for their spine problem, and if so, the type of care received, satisfaction with community-based care, and self-management strategies used, and
iii) Reported general health status, and community activities and gatherings.

The CR will provide an understanding of the type of spine care received (pharmaceutical and non-pharmaceutical care, referrals for imaging or treatment) in the 12 months prior to data collection. Pre- and post-implementation results will be contrasted to highlight any trends observed in spine care delivery.

### Implementation outcomes

All implementation outcomes will be reported descriptively. *Reach* will be reported as the proportion of partners (community leaders, residents) that engage with each of the implementation support sub-strategies, and local clinicians completing training and adopting the MoC and care pathway. *Adoption* (AIM, IAM and FIM total scores) and demographics will be reported, and associations of AIM, IAM, and FIM with other measures, such as characteristics will be assessed via Spearman rank correlations for continuous measures and Wilcoxon rank sum tests or Kruskal–Wallis tests for categorical measures. [85] *Adaptations* to the implementation support strategies will summarized using a modified version of a consistent coding framework of adaptations (FRAME) [89]. *Fidelity* will be reported as the number of people seeking spine care, provided each of the steps involved in triaging and/or using the spine care pathway were administered. The overall fidelity score will be calculated based on the number of people with spine symptoms seeking care across communities appropriately triaged based on the GSCI MoC. *Costing* will be include costs of providing the new clinical service and community movement program, training material,, visits to medical specialists, MRIs and CT scans, and cost related travelling expenses.

### Qualitative data analysis

As in the pre-implementation qualitative study, [52] all post-implementation interviews and focus groups will be audio recorded and transcribed verbatim. Coding and analysis will be managed using NVivo (QSR International, Version 12). Qualitative data analysis will be conducted through an interpretivist lens, [90] exploring participants’ experiences and thoughts. Two PhD students (NR, EB) will independently code each transcript guided by a mutual understanding of the TDF domain definitions and constructs within a domain, [63] and will meet weekly to review coding and achieve consensus. Two senior authors (AB, SM) familiar with the TDF will review the coded transcripts to solve any disagreements from the original coders to increase the reliability of coding (crystallization). Data will be analyzed using a combination of deductive and inductive coding. Deductive codes will be derived from the TDF domains, following a coding guideline to ensure consistency between coders. Data analysis will be carried out by pairs of trained RAs and two senior authors who will independently code the same subset of transcripts. [91] Coders will then meet after every 3–4 transcripts to discuss and reach consensus on code allocation, and the coding schemes will be refined and amended via an iterative process. The emergent coding tree will reflect both deductive and inductive codes [63, 91].

Key modifiable barriers identified will be mapped onto behavior change intervention techniques to inform the development and refinement of culturally acceptable implementation support strategies designed to support or change the health system to increase adoption of the evidence-based practice of the GSCI MoC into usual care [91, 92].

Study risk and risk mitigation strategies, and knowledge management and dissemination plans are presented in Additional files 1, Appendices 6 and 7 respectively.

### Implementation blueprint

With our partners, we will co-create an implementation blueprint to support high-value spine care (i.e., safe, timely, effective, efficient, equitable, patient-centred). [93] The 8-step process will be underpinned by implementation science frameworks that consider the multilevel and dynamic interactions between the interventions, the perspective and characteristics of diverse recipients (leaders, clinicians, patients, residents), the infrastructure, and the external environment (e.g., clinical guidelines): [12, 94, 95].

#### Engaging local partners

Relationship-building with the community is essential for sustainable development. [96] Prior to launching the study, a structured site visit took place to engage with the community leadership and an Elder. A collaborative research agreement and a data transfer agreement were signed in the summer of 2022 between interested parties.

Periodic site visits will be planned during the 3-year study period to (i) assess organizational issues (infrastructure requirements, partners and researchers roles and responsibilities, understand intake and flow of patients with spine symptoms, including GSCI MoC fit and acceptability); (ii) monitor service implementation and research activities and co-identify viable solutions with community partners; and (iii) assess how best to sustain the MoC and related support strategies.

#### Local context and population needs

In Canada, healthcare for Indigenous persons living on reserve in northern communities is managed federally. Under the universal healthcare plan, coverage for basic hospital and medical care at no charge to patients, but each province creates its own health insurance plan with some degree of variability across provinces. [97] In Manitoba, up to seven visits per year to a chiropractor are partially covered under the provincial health plan, but not under the federal health plan. Outpatient physical therapy is covered through an individual’s employment benefits. Both chiropractic care and physical therapy are healthcare services covered by Manitoba Public Insurance (motor vehicle accident injuries), and the Workers Compensation Board of Manitoba (workplace injury).

Cross Lake Nursing Station is managed by Health Canada, with resident nurses and general practitioner physicians as fly-in staff, providing essential care to community members. It has limited access to allied health care focused on spinal problems.

#### Selecting spine care model and interventions to meet needs

**Fig. 2:**
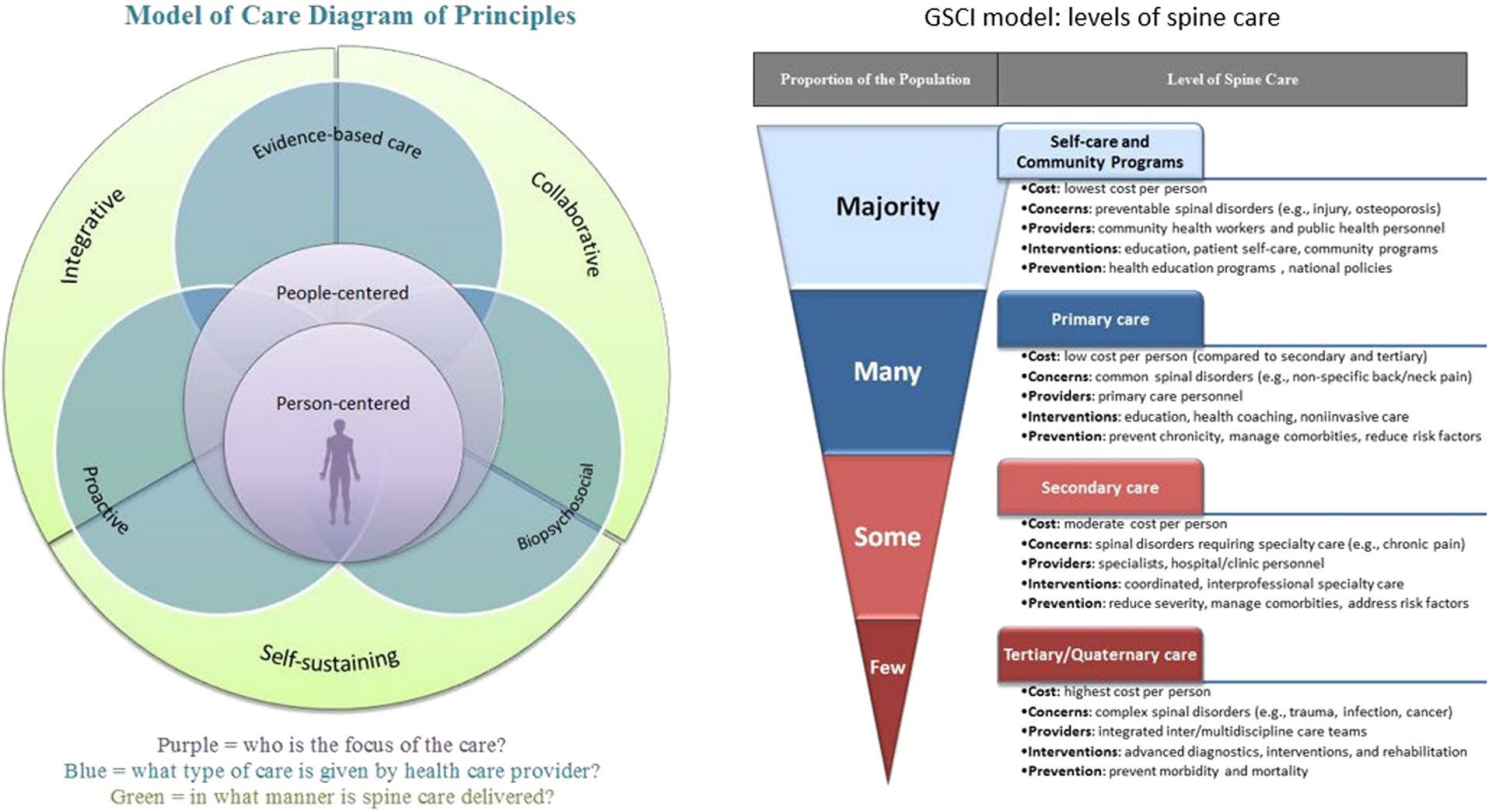
GSCI model of care and Levels of spine care

The GSCI MoC is a person- and people-centered, classification system and care pathway which considers the influence of cultural, economic, and healthcare system design elements. It identifies the resources needed to support the model’s delivery of care (Fig. 2). [16, 29, 98] This MoC provides a triage system and care pathways grouped into four specific categories of spine care aligned with high quality clinical practice guidelines: [99–102]

i) Community-based (e.g., education, reassurance, exercise programs, self-care);
ii) Primary care (community-based health care, providing screening for serious conditions, and ongoing accessible, comprehensive, evidence-based coordinated care);
iii) Secondary care (acute trauma and emergency care, imaging and diagnostic testing, surgical interventions); and
iv) Tertiary care (specialized medical and surgical care for complex spine problems).

These categories of care are to be integrated into the available healthcare system in partnership with local communities, existing healthcare facilities/providers, patients, and health policy makers (Additional file 1, Appendix 1. Implementation toolkit).

#### Gathering program materials

**Fig. 3:**
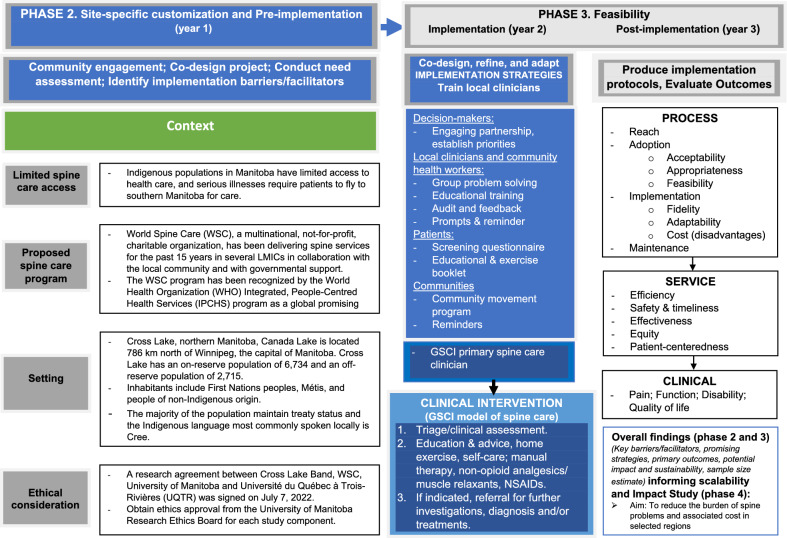
Logic framework (phase 2 and 3 studies)

Two logic models or road maps will be used to plan, execute, report, and synthesize the current implementation project: The administrative logic model provides an overview of the activities, output, and outcomes of the study (Additional file 1, Appendix 2); while the Implementation Research Logic Model (Fig. 3) presents the shared relationships among context, implementation strategies and process, service and clinical outcomes, allowing for the comprehensive specification of all introduced and present implementation strategies, as well as their changes (adaptations, additions, discontinuations) during the project [103].

#### Identifying implementation support strategies

Multifaceted strategies implemented in communities that are underserved and LMICs targeting infrastructure, supervision, other management techniques, training combined with group problem-solving can result in moderate to large practice changes [104]. Strategies targeting healthcare providers (e.g., educational training with clinician reminders and group problem-solving, practice facilitation, outreach visits, and input from local opinion leaders) and healthcare recipients (e.g., mass media interventions, self-management support, behavioural interventions and mobile phone text messaging) are generally effective in improving care. [104, 105] Increasing the frequency and the duration of strategies are likely to result in greater success and sustained practice change and better patient low back pain outcomes [106].

#### Comparing existing programs and culturally adapt processes

Additional file 1, Appendix 3 provides a detailed description of implementation support strategies. Following the Effective Practice for Organizational Change (EPOC) taxonomy of health systems interventions to expand equitable access to spine care, [107] we have provisionally selected eight implementation support strategies, along with 29 sub-strategies, targeting stakeholders across all levels to promote and sustain local interest in implementing the GSCI MoC (Table 3).

**Table 3:** Provisionally selected implementation support strategies (n = 8) and sub-strategies (n = 29)

**1. Health partnerships (year 1–3)** [108]
**1.1.** Partnership agreement signed by health districts or local government executive and local community leader
**1.2.** New or existing Local Implementation Team oversees program
**1.3.** The local Implementation Team is inclusive of a local clinician champion and community leaders to oversee the program and uses a self-assessment and action plan tool
**1.4.** Local implementation team meets weekly; macro level committees meet twice per year
**2. Health workforce capacity development: Local clinicians (year 2)** [55, 104, 105, 109–113]
**2.1.** Group problem solving (with or without formal teams) or collaborative improvement every 2–3 months **2.2.** Pre-service educational training: Local clinicians register and complete the 2 online educational training sessions at the beginning of year 2 **2.3.** On the job training: interactive workshop to ease knowledge integration, practice facilitation and educational outreach visits by a trained GSCI clinician overseeing patient encounters over 2–3 weeks using the Implementation Toolkit (Appendix 3, Table 1 and 3) **2.4.** Local clinician Champion training –1-day of face-to-face training session by trained a GSCI clinician, hosted by Local Implementation Team **2.5.** Peer coaching (improving routine supervision, benchmarking, or audit with feedback) by local champion for 12 months **2.6.** Weekly contact made with GSCI trained clinician via email and/or Zoom
**3. Health workforce capacity development: Community health workers (CHW) (year 2)–*Concurrent with item 2***
**3.1.** CHWs’ training –1-day of face-to-face training session hosted by trained GSCI clinician, hosted by Local Implementation Team, and facilitated by the local clinician Champions. Accommodation, meals and transport costs covered by the grant **3.2.** CHWs are trained to 1) recognize serious causes of spine problems via online or paper-based tools; and 2) deliver educational messages (reassurance, advice on self-care such as staying active, and basic exercise) for people with non-complicated spine symptoms/concerns (MoC class classes 1 through 3a, c and 4a) or to refer people with spine problems for further evaluation and treatments to local clinicians (MoC classes 3b, 4b and 5a, b and c). (Appendix 3, Table 2) **3.3.** Equipping and motivating CHWs to conduct outreach and referrals process from community to health centers **3.4.** Weekly contact made with in-community local clinician Champion via phone, email and/or face-to-face site visits for 12 months
**4. Educational tools to promote self-management (year 2–3)** [112, 114–120]
**4.1.** Self-administered online and paper format patient screening questionnaire to help make informed decision regarding the need to consult a licensed healthcare provider or to self-manage their spine pain (Appendix 3, Table 2) **4.2.** Online and paper format educational and exercise booklet; develop/adapt 1-page information resources (https://www.ccgi-research.com/patient-resources) **4.3.** Follow-up contact made by with in-Community Champion via phone, email and/or face-to-face site visits for 12 months
**5. Community movement program (year 1–3)** [105, 121, 122]
**5.1.** In-Community Champion training –1-day of face-to-face training session hosted by Local Implementation Team in Term 1: train the trainer on adult yoga-like mind–body classes, whereby Yoga instructors are trained on spine health, who, in turn, will deliver consistent messaging on spine health issues
**5.2.** Community support (community health education or social marketing of health services): spine health educational messages delivered monthly by CHWs through partnership with local community leaders at social gatherings (e.g., the village market or the church), clinic’s/healthcare centers, on social media platforms (Facebook via cell phones), using the local radio, and/or targeted at schoolchildren via their teachers **5.3.** Co-design and animate locally accepted community activity/exercise program
**5.4.** Follow-up contact made by local clinician Champion with in-community Champion via phone, email and/or face-to-face site visits for 12 months
**6. Resources (year 1–3)**
**6.1.** Printed posters outlining MoC Principles, triage system and care pathway to be displayed in the Nursing Station (Implementation Toolkit: Appendix 3, Table 1–2)
**6.2.** Equipment provided to support the delivery of MoC
**6.3.** Electronic resources housed on the program website (online) included: Overview of program presentation (Microsoft PowerPoint presentation) Project milestones to be achieved each term (over 3 years) Online quality training (GSCI videos), worksheet, peer observation materials Patient personal self-care plan templates Recess and lunch resources Policy templates Examples of community physical activity Tips and frequently asked questions
**7. Provision of prompts and reminders (year 2–3)** [113]
**7.1.** Weekly emails or phone calls made by the Local Implementation Team to local clinician and in-Community Champions to encourage implementation **7.2.** Automated or paper-based messages sent each term via the program website or hand delivered to Champions, local clinicians and CHWs to prompt completion of educational training modules/videos/booklet chapters and online (or paper-based) termly performance monitoring and feedback surveys
**8. Implementation performance monitoring and feedback (year 2–3)** [123]
**8.1.** Champions, local clinicians and CHWs complete all surveys via the program website or paper-based **8.2.** Feedback report sent to Champions, local clinicians and CHWs via email or hand-delivered

CHW, Community Health Worker; GSCI, Global Spine Care Initiative; MoC, Model of Care

Context-specific strategies are required for successful evidence implementation, and a number of common barriers can be addressed simultaneously using locally available, low-cost resources. [109] Guided by adaptation frameworks, [124–126] and in accordance with initial study findings and input from our partners, proposed support strategies will be modified, refined and culturally adapt to overcome implementation barriers or abandon [56, 127].

#### Adapting material for new context and monitoring

Prior to study onset, all relevant study material will be translated and culturally adapted following a 4-step process prior to being administered: (i) questionnaire adaptation/creation; (ii) expert team, partners, and local PIs (AB, SP) review; (iii) pre-testing of measures for readability and understandability; and (iv) data collection [128–130].

#### Co-refining the GSCI MoC and related support strategies with community partners

Using appreciative inquiry, [77] an affirmative approach to project evaluation shifting away from deficits-oriented evaluation methods towards a strengths-based or “desire-based” inquiry, [131] we will engage with community partners to discuss pre- and post-implementation study findings and promote self-determining further adaptions of the GSCI MoC and proposed implementation support strategies. We will deliberately choose to initially focus on factors that contribute to positive health care encounters through the discussion of experiences and best practices, and using that positive potential within participants, the community, and the wider system to create positive changes and commit to a way forward [77].

## Discussion

Spine pain is a highly prevalent and disabling, yet invisible condition. Major international clinical guidelines recognize that the vast majority of people with spine pain can be effectively managed with physical and psychosocial interventions, and discourage use of pain medication, steroid injections and spinal surgery. [99–102] However, the undertreatment of pain is systematically reported in the literature, particularly in marginalized populations. [39–41, 132–134] In addition, many health systems globally are not designed to support non-phamacological spine care approaches, with inadequate payment systems favouring medical care over patients’ self-management and rehabilitation, deep-rooted medical traditions and beliefs about care for spine pain, [135] and difficulty in accessing culturally safe and meaningful health care services for Indigenous peoples [43].

Promising solutions, practices and policies include providing accessible and culturally acceptable high-value spine care services, cultural safety training and trauma-informed care, addressing care providers’ biaises, and incentives to increase the number of Indigenous health care providers. [44, 45, 135] The reported findings of the two completed Phase 2 studies [51, 52] underscore the iterative nature of this research and provide critical data that will shape the subsequent implementation and evaluation phases. Results from this project are expected to further advance our understanding on the experiences and challenges of accessing spine care in a remote northern Indigenous community, and inform planning of a study aiming to determine the most effective means of sustaining and scaling the GSCI MoC to larger communities and to determine its personal, social and economic impact on underserved communities. To maximize the fit between the proposed spine care services, practice settings, and the broader ecological system, we will regularly engage with key partners and work toward reaching a mutual understanding throughout the project, seek to continually learn and problem solve, co-adapt implementation strategies with a primary focus on ongoing improvement considering multi-level contexts (e.g., culture, support, time, resources, funding) [136–138].

## Conclusion

Implementing the GSCI Model of Care in Cross Lake is expected to help reduce the burden of spine problems and related healthcare costs for the local community and serve as a scalable model for programs in other northern Canada regions and sites around the globe.

## Supplementary Information

Additional file 1.

## Data Availability

No datasets were generated or analysed during the current study.

## Abbreviations

CHS: Community health survey
CHW: Community health worker
CR: Chart review
EPOC: Effective practice for organizational change
FRAME: Framework for reporting adaptations and modifications-enhanced
GSCI: Global spine care initiative: https://www.globalspinecareinitiative.org/
IPCHS: Integrated, people-centred health services
LMICs: Low- and middle-income countries
MoC: Model of care
MRC: Medical research council
MSK: Musculoskeletal
PI: Principal Investigator
RA: Research assistant
REB: Research ethics board
SDG: Sustainable development goals
TDF: Theoretical domain framework
UK: United Kingdom
WHO: World Health Organization
WSC: World spine care: https://www.worldspinecare.org/
WSCC: World spine care Canada: https://www.worldspinecare.org/canada
