# Supplementary material for: Assessing the readiness and feasibility to implement a model of care for spine disorders and related disability in Cross Lake, an Indigenous community in northern Manitoba, Canada: a research protocol
Source: Chiropr Man Therap. 2025 Mar 13;33:12. doi: 10.1186/s12998-025-00576-1 (PMC11908001; doi:10.1186/s12998-025-00576-1)
Supplement: Supplementary file 1 — Additional file 1. [file 12998_2025_576_MOESM1_ESM.pdf]

World Spine Care

GSCI
Global Spine Care Initiative

Implementation toolkit

The Global Spine Care Initiative Model of Care Pathway Implementation Study

Phase 2 and 3

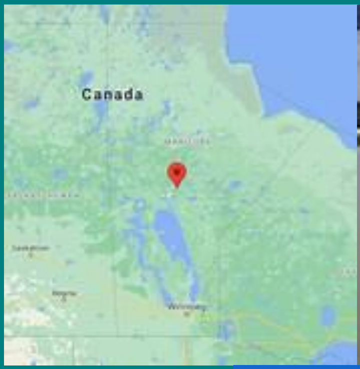

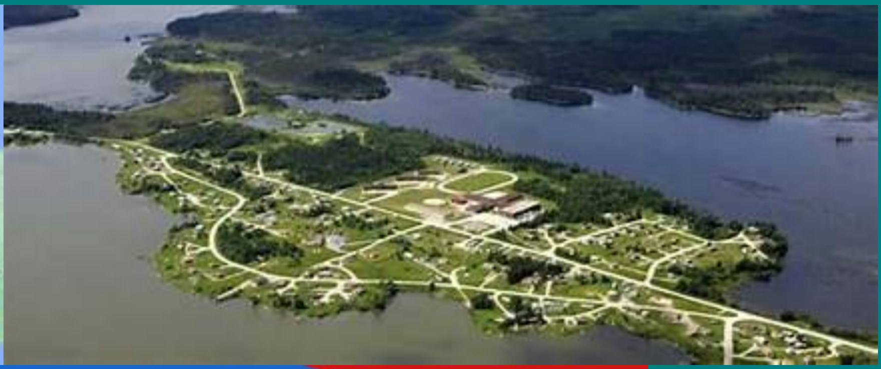
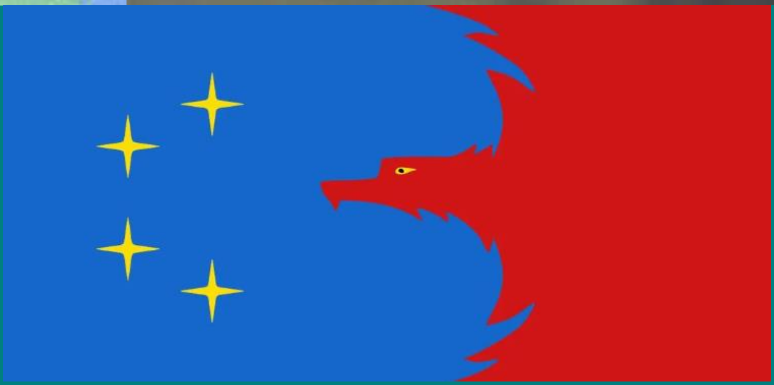

April 2024

TABLE OF CONTENTS

Appendix 1. Overview of the GSCI model of spine care…………….……….………**3**

Appendix 2. Administrative Logic Model - GSCI Spine Care Model ……….....……**4**

Appendix 3. Local Clinician referral form for people with spine symptoms and community health workers triaging form……………………………………...........….**7**

Appendix 4. Description of the proposed GSCI spine care services …...………**11**

Appendix 5. Description of Provisionally Selected Implementation Support Strategies ..………………………………………………………………………….…….**27**

Appendix 6. Risk and Risk Mitigation Strategies ………………..……………….….…**29**

Appendix 7. Knowledge Management and Dissemination……………………….**30**

Appendix 1. Overview of the Global Spine Care Initiative Model of Spine Care (MoC)

Within the Global Spine Care Initiative (GSCI) evidence-based model of spine care it is recognized that the great majority of people presenting with spine conditions (90% or more of all patients) can be appropriately managed through self-directed care and within the local primary care community. [96] Only a small percentage of individuals require advanced diagnostic or therapeutic interventions such as imaging, laboratory testing, referral to a medical specialist or surgery.

Building on this, the GSCI spine care pathway emphasizes creating access to community-based spine care and facilitating timely access to further diagnostics and treatment as needed. Licensed local primary clinicians (nurses, medical doctors, physiotherapists, chiropractors, doctors of osteopathy) or other CHWs who are trained in the MoC, will conduct screening and assessments to identify early on those individuals who can be safely and appropriately managed with primary spine care, provided in the local community. Health care institutions, dominated by the biomedical model, impose restrictions on Indigenous People's access to family and traditional rituals, which are seen as central to healing. The inclusion of traditional healing practices in a multidisciplinary pain treatment program would be beneficial, and the use of traditional medicine need not conflict with conventional treatments. [1] Primary spine care clinicians (chiropractor or physiotherapist) will provide the nonsurgical, minimal- or non-pharmaceutical care in Cross Lake Nursing Station. They will also facilitate referral for imaging or specialist opinions for individuals who present with more complex spine conditions or when an evidence-based intervention is not available at the primary spine care community level. Initial telehealth consults with an appropriate health care specialist from the secondary spine care regional hospital will be arranged. This can be followed up with in-person evaluation and treatment at a secondary or tertiary healthcare facility if needed.

The MoC contains two main components: 1) A classification system (triage/assessment/diagnosis) to help categorize people presenting with spine symptoms or concerns, and 2) A clinical care pathway to help guide care management (summarized in easy-to-use Flash Cards). Please refer to Appendix 4 of the Implementation Toolkit for an overview of the GSCI triage (classification) system designed for local clinicians (Table 1), Guide designed for CHWs and people with spine symptoms/concerns (Table 2), and Flash cards and wall chart explanations for clinicians at the primary, secondary, and tertiary levels (Table 3).

1.GSCI Classification system:

The initial clinical encounters will occur within the existing local health care facility or community centers by licensed local primary clinicians (nurses, medical doctors, physiotherapists, chiropractors, doctors of osteopathy) who will be trained in the GSCI MoC. Patients will be informed that they will have the opportunity to participate in the project and receive care as a study participant. No patients will be refused care based on whether they agree to participate or not. Patients will undergo an initial triage, clinical history and physical examination including completion of validated questionnaires on disability, pain levels and impact of their spine related symptoms on quality of life. Local clinicians will use this information to identify their spinal disorder according to the GSCI classification system and identify any co-morbid or unrelated health problems. The clinician will then consult the GSCI flash cards to determine which evidence-based care is appropriate and available at the community level. This may include education and reassurance, exercise prescription, specific evidence-based therapeutic interventions or referral for investigation and/or consultation for more complex spinal disorder classes.

2. GSCI Spine care pathway

If assessment and diagnosis results in the determination that the patient falls into GSCI classifications 1 through 3a, c and 4a [20] with the patients' informed consent, evidence-based non-invasive interventions will be offered by the primary spine care clinician based on the GSCI flash cards and widely accepted spine care guidelines. [97-99] This may include patient education and advice on directed self-care and exercise, non-pharmaceutical therapies (e.g. massage, acupuncture, manual therapy) and/or pharmaceutical therapies (e.g., simple pain medication and/or non-steroidal anti-inflammatory medication). A Glossary of Terms is available in Table 1. Patients not responding to locally available evidence-based care or who have red flags for serious neurological, systemic pathology progressive neurological deficit, spinal fracture or deformity, spine cancer or infection, serious systemic disease that place them into classifications 3b, 4b and 5a, b and c, will be referred to a secondary or tertiary centre/hospital associated for further investigation (laboratory tests, spine imaging) and/or spine surgery. [20] As people with spine symptoms often present with multiple conditions, interprofessional care will be facilitated according to patient needs. The end goal of care is to improve symptoms or prevent worsening of the condition, reduce associated disability and maintain optimal functioning. [100] These flash cards will aid clinicians in using the classification system and care pathway.

Appendix 2. Administrative Logic Model - GSCI spine care model

**Ultimate Outcome**	Improve access to high quality primary spine care services for adults with spine symptoms or concerns in Pimicikamak, northern Manitoba, Canada			
	↑	↑		
**Intermediate Outcomes**	1. Strengthened stakeholder engagement and improved delivery of equitable high-value spine care services among adult community members in Cross Lake		2. Increased capacity of adult community members to recognize, prevent and participate in the response to basic spine care needs	
	↑	↑	↑	↑
**Immediate Outcomes**	100 Effective linkages and improved communication created between Nursing Station local clinicians, Cross Lake health services, and referrals centres in Winnipeg	200 Increased local clinicians' knowledge and skills on spine care and extend services from training program	300 Increased capacity and empower adults with spine symptoms or concerns to understand spine care issues, access primary spine care, and self-manage	400 Strengthened engagement and increased capacity of community residents to participate in prevention program
**Cross-cutting outcome**	Data and evidence generated about the clinician and community readiness to implement the model of spine care (MoC); feasibility (i.e, recruitment, adherence and retention rates) to implement the MoC; and stakeholder's intention to continue to support the new spine care service and community movement (educational) program after the study.			
	↑	↑	↑	↑
**Outputs**	110 MOU signed between local community leadership and WSC, thus offering comprehensive support	210 Local clinicians (GPs, nurses) are trained on GSCI spine triage and care pathway (including rehabilitation interventions)	310 Community health workers provide educational material (online, education booklet) on educational (movement) program to people with spine symptoms or concerns	410 Adapted GSCI and previously designed educational materials, targeting community key issues related to spine heath, self-management strategies and opioid use.
	120 Band Council representatives have a clear understanding about the importance of their role in helping raise awareness and improve spine health	220 Identify and train community health workers (CHWs) on recognizing serious causes of LBP (i.e. red flags), referral process, and providing self-care support strategies	320 People with spine symptoms or concerns are trained on initial GSCI classification using simple triaging questions to help make informed decision regarding the need to consult a care provider in the	420 Community residents are aware of educational (movement) program, self-management strategies, and spine care services

			future or to self-manage their spine symptoms	
	130 Project is endorsed by the Health District representative	230 Community health worker equipped and motivated to conduct outreach and provide referrals linking community to health centres		430 Community residents use self-administered (online or paper format) patient screening questionnaire to help make informed decision regarding the need to consult a care provider or to self-manage their spine pain
	140 Quarterly and annual review meetings conducted with Community Leadership and Health Director	240 Local clinician Champions (GP and Nurse) orientated on supervision of peers		440 Community awareness campaigns delivered on spine health via local radio and/or TV
	150 Research staff (research assistant; graduate students; research coordinator, local staff) trained on data collection, documentation, descriptive analysis and reporting	250 Community health worker Champion trained as resource person for community educational program		450 Community educational (movement) programs are regularly attended
		260 Quarterly and annual review meetings conducted with healthcare team		460 Quarterly and annual review meetings conducted with village partner
	↑	↑	↑	↑
**Activities**	111 Obtain support from local community leaders, health district, and local government for the project	211 Train local clinicians on GSCI triage system and care pathway (i.e. flash cards including the delivery of evidence-based spine care and referral process)	311 Design/adapt educational booklet and materials, targeting self-care strategies and opioid use	411 Culturally adapt educational (movement) program and train instructors on spine health issues
	121 Hold meeting to sensitize and orient community leaders on the importance of their roles in helping raise awareness and improve spine health	221 Train local clinicians and community health workers deliver educational messages, reassurance, advice to stay active, and basic exercise for people with spine symptoms or concerns	321 Train community health workers to conduct outreach and on referrals linking community to health centres	421 Organize spine health awareness campaigns targeting community key issues related to spine heath, self-care strategies, and opioid use

	131 Leaders meet with government representatives to obtain support / endorsement	231 Trained local clinicians and research staff document clinical encounters, use referral system and link with health centres where appropriate	331 Translate and culturally adapt self-administered patient screening questionnaire to help make informed decision regarding the need to consult a care provider or to self-manage their spine pain	431 Translate/culturally adapt, and conduct orally administered community survey (pre-implementation)
	141 Facilitate quarterly and annual review meetings of the project with project stakeholders in the three countries	241 Conduct an orientation session with local providers on supervision of community health workers	341 Culturally adapt, and conduct pre- and post-implementation chart reviews	441 Translate/culturally adapt, and orally administer adoption survey questionnaires (post-implementation)
	151 Translate/culturally adapt interview topic guides, and conduct pre- and post-implementation interviews	251 Translate/culturally adapt interview topic guides, and conduct pre- and post-implementation focus groups	351 Culturally adapt and orally administer Community Health Survey (pre-implementation)	451 Translate/culturally adapt interview topic guides, and conduct pre- and post-implementation interviews
	161 Administer adoption survey questionnaires (post-implementation)	261 Administer adoption survey questionnaires (post-implementation)	361 Translate/culturally adapt interview topic guides, and conduct pre- and post-implementation interviews	

Appendix 3. Description of Provisionally Selected Implementation Support

Based on existing literature and initial stakeholder consultation across study sites, we have provisionally selected 8 implementation support strategies. Critical elements in promoting and sustaining local interest in implementing the MoC and spine care pathway include the following:

**1. <u>Health Partnerships at all ecological levels</u>**<br><br>While clinical care and health service delivery organisations tend to focus on the 'micro' and 'meso' levels (our end-users), implementation of MoCs for musculoskeletal disorders must be supported by policy and financing models that emphasize first-line, effective intervention. [2] This macro level includes policy and governance, information systems and strategy to deliver health services. Further, the 'global' level should consider the changing health systems in the 21st century facing new and complex challenges such as rapid population ageing, increasing disability attributed to non-communicable diseases and associated multimorbidity of NCDs. [3] Applying principles and using resources outlined in the International Health Links Manual, [4] we have established strong partnerships at the micro, meso and macro levels, and at the global level to provide guidance on the project. Tasks involve: [5]<br>• 1.1: Partnership agreement signed by health districts or local government executives and local community leaders;<br>• 1.2: New or existing Local Implementation Teams oversees program;<br>• 1.3: The local Implementation Team is inclusive of a local clinician champion and community leaders to oversee the program and uses a self-assessment and action plan tool;<br>• 1.4: Local Implementation Team (micro/meso levels) meet once per month; macro and global level committees meet twice per year.
Micro level (end-users): Refers to where the action takes place (i.e. settings where the MoC is implemented in the communities and service delivery levels (onsite primary spine care for most people with spine symptoms or concerns, and patient referrals to distant community health centres or hospitals for secondary or tertiary spine care). ***Meso level*** refers to those involved in managing services, running clinics, who have to consider resources needed. Through regular meetings over the preceding months leading to the grant submission, the Local Implementation Team has established strong collaboration and sought input from partners (local governing bodies and health care clinicians and managers) on the various parts of the research process. The Local Implementation Team in each country will oversee the activities and decisions impacting local clinicians, community health workers, people with spine symptoms or concerns, and the communities throughout the project.
Macro level (stakeholders): Refers to everything surrounding a community or organization; this can include the health system regional or national environments. The launching and sustainability of the project will require ongoing support from governments and local health districts. For instance, chiropractic services should be maintained as part of the Nursing Station to sustain spine care delivery in Cross Lake. Advisory groups concerned with health service delivery organisations such as community health centres and other service delivery organisations will help operationalize the model of spine care. Possible benefits for macro level stakeholder collaborators include added visibility from media coverage (message showing how they do more for their communities), and helping address important care needs (message highlighting how the proposed evidence-based solution can help address this high burden problem).
Global level (partner organizations): Refers to well established international organizations aiming to improve care to people with musculoskeletal disorders, including spine symptoms around the world. Several WSC researchers and clinicians took part in WHO guideline for non-surgical management of chronic primary LBP in adults in primary and community care settings, released in December 2023. [6] These recommendations aligned well with the GSCI spine care model and the online educational training modules. Support/endorsement from the WHO for this project may facilitate both implementation and sustainability. Further, this is congruent with government initiatives to support activities in northern Manitoba.

2. <u>Health workforce capacity development to improve spine care delivery</u>: *Local licensed clinicians*

Improved health care provider performance should lead to stronger health systems and better health outcomes for individuals and populations. [7] Promising strategies to improve the skills and knowledge of local clinicians in LMICs include online educational training, practice facilitation, educational outreach, local opinion leaders, audit and feedback, and tailored interventions, and reminders. [8-10] Training will consider sex, gender and age-related differences in spine pain risk factors, diagnosis, care seeking behaviour, management, and prognosis. [11] We will use the Musculoskeletal (MSK) Core Capability Framework for first point of contact practitioners [12] to help identify training and development needs of the healthcare workers, and train local clinicians and HCWs to deliver spine care. The MSK Core Capability Framework comprises 70 specific capabilities mapped to 14 broader capability areas, summarised into four domains: Person-centred approaches; Assessment, investigation and diagnosis; Condition management, interventions and prevention; and Service and professional development.

1) *Group problem solving* (with or without formal teams) or collaborative improvement can lead to improved practice when combined with other approaches. Aligned with the participatory approach, [13] end-users will meet regularly throughout their training and thereafter, to discuss the value of and need for the MoC, identify their own strengths in triaging and managing people with spine symptoms or concerns, and be empowered to provide and share possible solutions to increase the chances that the MoC implementation will be acceptable, applicable, feasible, and adopted in routine clinical practice.

2) *Pre-service educational training:* There is a need to provide health care practitioners engaged in non-surgical care with an interprofessional harmonised and evidence-based curriculum on patient-centred collaborative care. [14, 15] Interested local care providers will be invited to online modules aimed at developing expertise in spine management and empowering care providers. Local clinicians will be asked to review (asynchronous) four educational modules which train on the GSCI MoC and specifically how these apply to their local clinic setting. Case studies are reviewed and a quiz will be provided.

3) *Interactive workshop, practice facilitation, and educational outreach visits* (i.e., on-the-job training with face-to-face visits to individual HCPs at their workplace after online training) and training that incorporate clinical practice and training at health care providers' work site appear to be effective. [16] Educational outreach visits by a GSCI primary spine care clinician to provide required knowledge and skills to assess, triage/classify and manage people using the evidence-based GSCI spine care pathway within culturally acceptable boundaries, will be undertaken. Following the recommended approach by Kongsted et al. (2021), there will be a strong focus on how best to deliver self-management support interventions to equip patients with skills to actively participate in their own spine care, and take responsibility for the management of their chronic spine condition. [17]

4) *Local clinician Champion training* –1-day of face-to-face training session by trained GSCI clinician, hosted by Local Implementation Team.

5) *Peer coaching* (improving routine supervision, benchmarking, or audit with feedback) by a trained Champion (and the GSCI primary spine care clinician) are recommended approaches. [8] Once trained in the MoC, quality assurance will be maintained with monthly calls involving the local clinicians and a designated clinical advisory team.

6) *Mobile phone messaging reminders* or *manually-generated paper reminders* to improve clinician adherence to the MoC (triaging and flash-cards)

3. <u>Health workforce capacity development to improve spine care delivery</u>: *Community health workers (paid, volunteers) including village nurses; traditional healers*

Financial incentives for health-care providers appear to have modest to moderate effects. However, combining training of community health workers with financial support is likely to be more effective than training alone. [7]

Training community health workers facilitated by local clinician Champions, will consist of:

1. Using the Community Health Worker Guide to recognize serious causes of spine symptoms by asking standardized questions (Appendix 3. Table 2. Community Health Worker Guide)

2. Delivering educational messages (reassurance, advice on self-care such as staying active, and basic exercise) for people with non-complicated spine problems (MoC class, classes 1 through 3a, c and 4a) or to refer people with spine problems for further evaluation and treatments (MoC classes 3b, 4b and 5a, b and c).

3. Equipping and motivating community health workers and village nurses to conduct outreach and referrals process from community to health centres.

4. Weekly contact made with in-community local clinician Champion via phone, email and/or face-to-face site visits for 12 months.

4. <u>Educational tools to promote self-management in people with spine symptoms or concerns</u>

Self-management refers to an individual's ability to manage the symptoms, treatment, physical, and psychosocial consequences and lifestyle changes inherent in living with a chronic condition. [18, 19] Patients and families consistently, across all illnesses, prioritize self-management support as a means of empowerment in order to manage their health, [20] however, many do not know where to search for needed information and "don't know where to start" to accomplish all the lifestyle changes recommended by their health care providers. The goal of self-management is for individuals to achieve the highest degree of functioning and lowest level of symptoms despite the severity of the condition. Achieving this however requires providing people with the right information and behavior change support given their level of disability. [17]

Promising self-management support strategies targeted at healthcare recipients include interventions to increase uptake of screening (education, counselling, access to health promotion nurse and intensive recruitment), intensive self-management support and adherence; reminders and mobile phone text messages. [9]

Self-management support includes actions related to intervention planning, delivery, and evaluation. [17] Patient education will include evidence-based self-management strategies, advice and education for pain prevention, minimizing pain, stress, depression and anxiety (which often accompany chronic pain). [21-23] Further, key aspects of self-management such as collaborative goal-setting and shared decision-making, monitoring of behaviors and symptoms together with regular feedback from providers to shape health behaviors are rarely integrated into current models of care. [24] We will use patient education materials (paper-based and online), static and interactive, that are being used as part of interdisciplinary spine pain management programs. We will:

1. Develop and refine a self-administered online and paper format patient screening questionnaire to help patients make informed decisions regarding the need to consult a care provider or to self-manage their spine pain (Appendix 3, Table 2. Community Health Workers Guide);
2. Provide an online and paper format educational and exercise booklet; [25] develop/adapt 1-page information resources (https://www.ccgi-research.com/patient-resources);
3. *Send manually-generated paper reminders* or *mobile phone messaging reminders* to promote adherence to recommended self-care strategies. [9, 10]

5. Community educational movement program to raise awareness about spine health

Community support (e.g., community health education or social marketing of health services) can help increase contextual and implementation diversity. Combined with health-care provider training, community support results in larger effect sizes. [7, 9]

1. Spine health educational messages delivered monthly by GSCI trained clinicians and/or healthcare workers (village nurses; community health workers) through partnership with local community leaders at social gatherings (e.g., the village market or the church), at clinic's/healthcare centre's, on social media platforms (Facebook via cell phones), [26] using the local radio, and/or targeted at schoolchildren via their teachers;
2. Locally accepted community movement/exercise program whereby we will train local instructors on spine health, who in turn will deliver consistent messaging on spine health issues.

6) Resources and educational material

Printed posters outlining MoC Principles, triaging and spine care pathway will be translated and displayed in community clinic/health centers. Equipment provided to support the delivery of MoC may include electronic resources housed on the program website (online) such as overview of program presentation (Microsoft PowerPoint presentation), project milestones to be achieved each term (over 3 years), online quality training modules and GSCI Community Health Worker (CHW) Guide: Triaging people with back or neck symptoms or concerns, training videos, and patient personal self-care plan templates, examples of community physical activity, and tips and frequently asked questions.

7) Provision of prompts and reminders

Weekly emails or phone calls will be made by the Local Implementation Team to local clinician champions (self-identified or via the primary spine care clinician) and in-Community Champions (nominated by community leaders) to encourage implementation. Automated or paper-based messages sent each term via the program website or hand delivered to Champions, local clinicians and CHWs to prompt completion of educational training modules/videos/booklet chapters and online (or paper-based) termly performance monitoring and feedback surveys.

8) Implementation performance monitoring and feedback

We will apply the principles of capability review to demonstrate clinicians' fulfilment of the MSK Core Capability Framework. Assessment may take the form of case-based presentations, theoretical and/or practical tests of knowledge, skills and behaviours, critical reflections on practice, and portfolios that provide of evidence of learning. [27] Further, Champions and CHWs will be asked to complete all surveys via the program website or paper-based. Feedback reports will be sent to Champions, local clinicians and CHWs via email or hand-delivered.

Appendix 4. Table 1. Local Clinician Guide: Overview of the triaging system including standards (essential and desirable) required of program [28]

Step 1 Awareness

An individual becomes aware of a spinal symptom or concern and decides to seek information and whether or not to seek professional care.

Step 2 Initial Triage

The initial step of triage is done by the individual or may be done by a health care worker or provider. A screening set of questions identifies the nature, onset, severity of symptoms, presence of neurological symptoms and presence of serious or systemic pathology and places the concern into one of six classes. Class should be determined for each spinal region.

Class 0	Class I	Class II	Class III	Class IV	Class V
No or minimal symptoms	Mild symptoms	Moderate or severe symptoms	Neurological symptoms	Spinal fracture or deformity	Serious or systemic disease

Step 3 Provider Assessment

A health care provider performs further triage and assessment (detailed history, examination, and appropriate diagnostic tests) for each region and concern. The assessment determines the subclass for each spinal region.

Class 0a	Class 0b	Class Ia	Class Ib	Class IIa	Class IIb	Class IIc	Class IId	Class IIIa	Class IIIb	Class IIIc	Class IVa	Class IVb	Class Va	Class Vb	Class Vc

Step 4 Intervention

Interventions are matched to the findings from the assessment. Before starting any intervention, the clinician, patient, and any caregivers review the benefits, harms, costs and availability of the recommended treatment options and make a shared decision on the treatment to be initiated. Once agreed, the treatment is implemented.

Step 5 Outcomes

After the intervention has been implemented, there are three general outcomes.

1. Symptoms or concerns are resolved. The individual is discharged (e.g., continue self-management)
2. Symptoms or concerns are ongoing. Further clinical history or examination, including consideration of compliance with recommended treatment, and additional testing are considered. This may result in confirming or revising the class and subclass and revisiting the care pathway for recommended intervention options.
3. Person is non-responsive, worsening or has chronic symptoms despite having completed the intervention. Referral to other provider or inter/multidisciplinary care as described in the care pathway.

Table 2. Clinician referral form for people with spine symptoms /concerns

World Spine Care

SPINAL TRIAGE / REFERRAL FORM*

Name: DOB:
Address:

WSC Clinic ____________________
Contact person: ____________ Phone: ____________

Presenting Spine Symptoms

Location	Pain severity + Activity limitations	Onset	Progression
☐ Low back	☐ No or minimal pain; no limitations	☐ New Onset (<1 week)	☐ Non-progressive (stable)
☐ Mid back	☐ Mild pain; mild limitations	☐ Acute (<3 months)	☐ Rapidly progressive
☐ Neck	☐ Moderate pain; moderate limitations	☐ Chronic (>3 months)	☐ Slowly progressive
	☐ Severe pain; severe limitations		☐ Unknown

° see reverse for pain scales

TRIAGE STEPS		ACTION STEPS
**Step 1:** Suspected serious or or systemic pathology impacting or causing spine symptoms (e.g., cancer, infection [TB, AIDS] Inflammatory joint disease)? Class Va,Vb,Vc	If YES →	❑ Referral for medical or specialist care to rule out **serious disease** impacting spine pain symptoms. • May need immediate care, imaging and laboratory testing
**If NO** ↓		
**Step 2:** Suspected fracture or dislocation (e.g., recent severe trauma, osteoporosis? Class IVb	If YES →	❑ Referral for imaging or other testing to rule out **fracture or dislocation**. • May need orthopedic surgery consultation
**If NO** ↓		
**Step 3:** New onset, severe or progressive spinal cord or cauda equina syndrome (e.g. muscle weakness, bowel or bladder dysfunction, loss of balance)? Class IIIb	If YES →	❑ Emergency referral and MRI (CT if MRI not available) to rule out **spinal cord compression or cauda equina syndrome**. • May need emergency surgery
**If NO** ↓		
**Step 4:** Presence of moderate or severe spine pain with or without radiation into arms or legs (Class IIa, IIb, IIc, IId), with or without non-progressive numbness, tingling or muscle weakness (Class IIIa, Class IIIc), stable deformity (Class Iva)?	If YES →	❑ Refer to World Spine Care clinic on a priority basis
**If NO** ↓		
**Step 5:** Mild or minimal uncomplicated spine pain? Class 0b,1a,1b	If YES →	❑ Provide education and advice on self care, self-directed exercise, information on risks factors<br>❑ Refer to community-based program<br>❑ Referral to World Spine Care clinic

REFERAL TO WORLD SPINE CARE CLINIC

Priority status: ☐ Urgent ☐ Non-urgent **Patient is aware of referral:** ☐ Yes ☐ No

Diagnosis: ____

____

Psychosocial factors/Medications: ______________________________

Services requested: :______________________________________

Referring Provider: ________________________ Signature: ________________

Designation: ____________________________ Date: ____________________

Primary Spine Care Clinician use only
Date referral received: ______________ *Date of Initial assessment:* ______________

*GSCI MODEL OF CARE (2023). Source: Haldeman et al., EuroSpine J (2018) 27(Suppl 6): S901-914

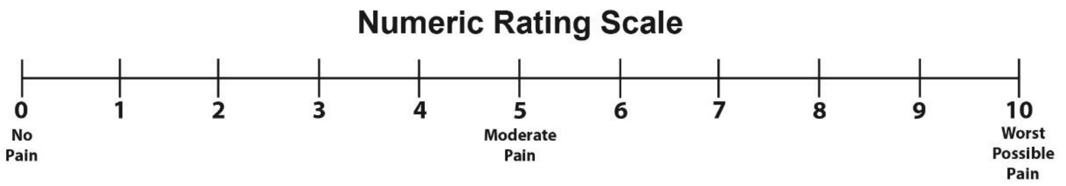

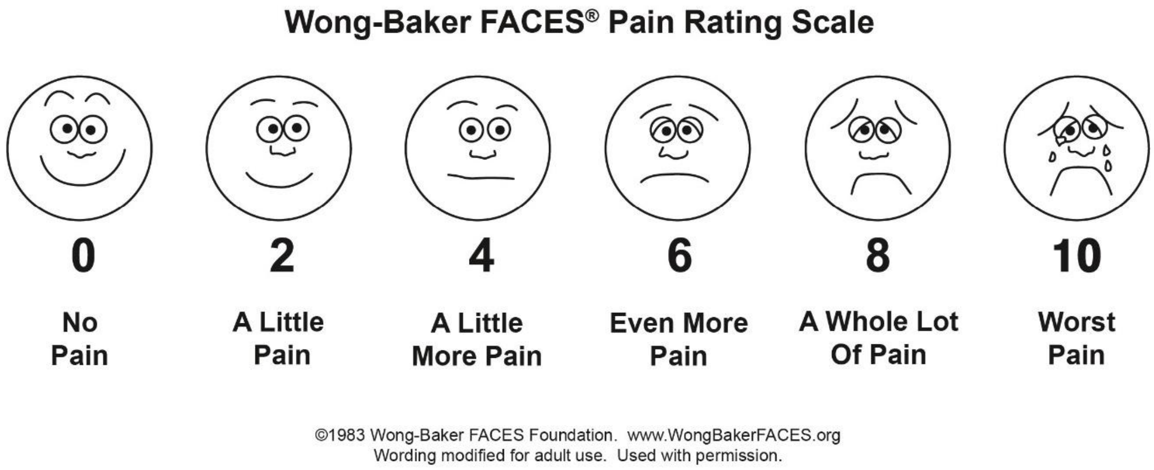

WORLD SPINE CARE - GSCI classification system

**Class 0:** No or minimal symptoms	0a No evident risk factors for a spine-related disorder or pain<br>0b One or more risk factors for a spine-related disorder or pain
**Class I:** Mild symptoms	Ia Acute or subacute, mild pain<br>Ib Chronic or recurrent, mild pain
**Class II:** Moderate or severe symptoms	IIb Chronic or recurrent, moderate pain<br>IIa Acute or Subacute, moderate pain<br>IIc Acute or subacute, severe pain<br>IId Acute or subacute, severe pain
**Class III:** Neurological symptoms	IIIa Minor non-progressive<br>IIIb Acute, major and progressive<br>IIIc Chronic and stable
**Class IV:** Spinal fracture or deformity	IVa Stable spine pathology, no correlation with symptoms<br>IVb Acute or chronic spine pathology, correlation to symptoms
**Class V:** Serious or systemic pathology	Va Severe, acute spinal pathology, requires immediate intervention (emergency)<br>Vb Severe, slowly progressive spinal pathology, requires intervention (non-emergency)<br>Vc Spine symptoms originating from non-spine pathology, requires immediate intervention (emergency)

Table 3. Local clinician guide: GSCI Spine Care Pathway (Flash Card and Wall Chart explanation). [28, 29]

The GSCI Flash Cards and Wall Charts are extracted from the GSCI Care Pathway and Model of Care articles. They should be considered decision aids for patients with spine-related symptoms and for clinicians from whom they may seek care.

First set of 3 cards are a Patient Guide - Self Assessment and Decision Aid

I have back or neck pain, what questions should I ask myself before I decide what to do?
This can be done personally or with the help of a health care clinician (nurse, traditional healer, family physician, chiropractor, physiotherapist) or first responder (ambulance, police)

Card 1: What are my symptoms? 5 questions
Card 2: What Class of spinal disorders do I have? 6 classes
Card 3: Management decision Guide for each Class of spinal disorders

This information should be reviewed by the spine care clinician at the first visit and before a detailed history and examination is initiated.

Second set of 3 cards are a Clinician Guide – Initial Assessment and Reassessment

Card 1: Initial Assessment: essential questions from history
Card 2: Determination of Class and Subclass of Spinal Disorders. Confirmed by a detailed history and examination
Card 3: Reassessment: subsequent visit outcomes, questions and decision options

Third set of 7 cards are a Clinician Guide for Treatment Interventions

These clinical decision aids should be considered options or recommendations based on the best available evidence. The clinician and patient should decide, together, which treatment option fits reasonably with available resources and patient preferences. The treatment decision should be guided by and is dependent on the training, experience and judgement of the clinician.

Card 1: Class 0 a,b (Minimal or no symptoms)
Card 2: Class I a,b (Mild Pain)
Card 3: Class II a,b (Moderate pain)
Card 4: Class II c,d (Severe pain)
Card 5: Class III a,b,c (Neurological symptoms or signs)
Card 6: Class IV a,b (Spine trauma or deformity)
Card 7: Class V a,b,c (Red flag serious spine pathology)

I. Patient Guide – Self Assessment

I have back or neck pain, what questions should I ask myself before I decide what to do?

Card 1. What are my symptoms?

Answer the following 5 questions to determine whether it is necessary to seek professional help, what kind of health care practitioner to see and how quickly.

This can be done personally or with the help of a health care clinician (nurse, traditional healer, family physician, chiropractor, physiotherapist) or first responder (ambulance, police)

1. **What am I feeling?**
   - a. no or minimal discomfort b. mild pain c. severe pain
   - d. numbness or tingling e. muscle weakness f. loss of balance
   - g. new onset of bladder or bowel problems like loss of control

2. **Am I feeling pain beyond my spine?**
   - a. no. b. down the legs c. down the arms
   - d. new or different headaches e. chest pain

3. **Are the symptoms stopping me from doing my normal activities?**
   - a. no. I can do everything
   - b. yes, a little. I can do most activities
   - c. yes, a lot. I have difficulty doing anything

4. **Have I had a serious accident or fall?**
   - a. no b. yes

5. **Do I have or have had any other serious diseases?**
   - a. no b. yes cancer
     infection such as TB, HIV;
     osteoporosis, steroid use, age over 60
     inflammation of my joints or rheumatoid disease
     serious neurological disease

Card 2. What Class of spinal disorders do I have?

Class 0 - Yes on 1a and no on all other questions

Class I - Yes on 1b and no on all other questions

Class II - Yes on 1c and 3c and no on all other questions

Class III - Numbness, tingling in arms or legs, muscle weakness, new onset of bowel or bladder problems (Yes to 1d, 1e, 1f, or 1g), severe new onset of headaches or chest pain (yes to questions 2b, 2c, 2d, or 2e)

Class IV - Severe fall or accident with severe spine pain (yes on 1c and 4). Suspect a spinal fracture

Class V - Yes on any of the conditions noted in question 5.

Card 3. Now that I know which Class of spinal disorders I have, what do I do?

Class 0 - Seek information from a reliable source (consider the WSC/GSCI article/website on risk factors)

Class I - Consider self-care, recognize that mild spine pain is very common, usually does not become disabling and improves or resolves over time without treatment

If the symptoms persist for a prolonged period, consider seeking the care of a spine care clinician who is knowledgeable about spinal disorders. Recognize that testing such as x-rays and MRIs rarely help in the decision as to which treatment to consider. Referral to a surgeon is not necessary.

Class II - Recognize that it is common for even severe spine pain to improve without requiring x-rays or other testing or treatment. If the pain does not resolve, is due to trauma or the pain is intolerable it may be necessary to see a spine care clinician to consider testing and to provide symptomatic relief

Class III - If recent onset (less than one week), consider immediately seeking a health care practitioner (primary spine care, chiropractor medical physician or emergency room) for a detailed examination. May require x-rays, MRI or other testing and treatment

Class IV - Keep still if due to trauma. Do not move and have someone call for an ambulance.

Class V - Seek care from your medical physician or specialist to determine whether the serious disease is causing your spine-related symptoms

II. Clinician Guide – Initial Assessment

Card 1. Essential questions from history

The following questions determine the GSCI Class and Subclass.
These questions should be asked before considering diagnostic testing or treatment

1. **Where in the spine does the person have symptoms or concerns?**
   Low back   Mid- back   Neck

2. **What are the symptoms or concerns for this spine region?**
   - ☐ **minimal discomfort (pain 0-1/10)**
   - ☐ **mild (pain 2-4/10)**
   - ☐ **moderate (pain 5-7/10)**
   - ☐ **severe (pain 8-10/10)**
   - ☐ **numbness or tingling**
   - ☐ **muscle weakness**
   - ☐ **loss of balance**
   - ☐ **new onset of bladder or bowel problems like loss of control**
   - ☐ **deformity, scoliosis**
   - ☐ **dysfunction, inability to perform daily activities**
   - ☐ **other spine-related concern**

3. **How long has the person had the symptoms?**
   a. less than 3 months   b. more than 3 months

4. **Do pain or neurological symptoms radiate beyond the spine?**
   a. No.   b. Yes. Legs, arms, head, chest

5. **How much do symptoms cause difficulty with normal activities?**
   a. **None**
   b. **Patient can do most but not all normal activities.**
   c. **Patient has difficulty doing most activities**

6. **Has the patient experienced a recent trauma, such as a serious accident or fall?**
   a. No   b. Yes

7. **Is there a history or risk of serious systemic disease, cancer, or infection?**
   a. No   b. Yes: cancer, infection such as TB, HIV, osteoporosis, steroid use, and/or age over 60, inflammatory or rheumatoid joint disease, serious neurological disease

Card 2. Determine Class and Subclass of Spinal Disorders

The clinician completes the questions above and conducts a detailed history and examination to ensure that the patient's complaints are confirmed and consistent with the initial impression.
The Class and Subclass should be determined for each spine related symptom

Class 0

No symptoms or only minimal discomfort (yes on 1a) and no on all other questions but has concerns about possible future spine problems.

Subclass considerations:

- **Class 0a = no history of risk factors**
- **Class 0b = history of risk factors**

Class 1

Mild pain or discomfort (yes on 1b). No to all other questions.

Subclass considerations: Question 3

- **Class Ia = a*cute* (duration < 3 months)**
- **Class Ib = c*hronic* (duration > 3 months)**

Class II

Moderate or severe pain (yes on questions 2c or 2d) and question 5 (moderate or severe activity difficulties). No on all other questions.

Subclass considerations: Question 3 and question 5

- **Class IIa = *acute* (duration < 3 months) *moderate* pain and disability**
- **Class IIb = c*hronic* (duration > 3 months) *moderate* pain and disability**
- **Class IIc = *acute*, *severe* pain**
- **Class IId = c*hronic, severe* pain and disability**

Class III

Neurological symptoms or deficits (yes on 4 or 2d, 2e, 2f, 2g)

Subclass considerations:

- **Class IIIa = *minor or non-progressive***
- **Class IIIb = *acute, major or progressive***
- **Class IIIc = *chronic and stable***

Class IV

Severe structural spine pathology such as acute fracture (yes on question 6 and 2c or 2d on question 2) This Class also includes old or asymptomatic structural pathology (yes on question 2h) that can be noted clinically or on imaging.

Subclass considerations:

- **Class IVa = *stable* spine structural pathology with no serious symptoms or red flags**
- **Class IVb = *acute* (e.g. fracture) *or chronic* (e.g. instability) spine *structural pathology* which correlates with symptoms**

Class V

Spine related symptoms due to *serious systemic pathology* (yes on any of one of question 7) confirmed by more detailed history and clinical or laboratory examination

Subclass considerations:

- **Class Va = *severe acute spine pathology*. Requires immediate attention (emergency).**
- **Class Vb = *slowly-progressive spinal pathology*. Requires intervention (non-emergency)**
- **Class Vc = *symptoms originating from non-spinal pathology*. This is an emergency.**

Clinician Guide – Reassessment

Card 3. Outcomes Assessment and follow-up decision options

The following 5 outcomes are possible following an intervention. For each of these outcomes specific clinical decision steps should be considered.
Outcomes should be considered for each spine-related symptom

1. **Symptoms have resolved or are no longer a problem**
   a. Re-assess to confirm the history
   b. Ask for and answer any patient questions or concerns
   c. Discharge following reinforcement of education messages on risk and prognostic factors for future symptoms

2. **Symptoms have improved, intervention appears to have helped but there is still a problem. Requesting additional care**
   a. Follow-up pain and disability questionnaires and limited examination to confirm a positive response to current intervention
   b. Confirm whether the patient is in the same or a less severe Class and Subclass in the GSCI Classification
   c. Continue with the same or an alternative intervention from the list of options for interventions within the current Class and Subclass

3. **No change in symptoms and intervention has not been effective. Not any worse.**
   a. Re-examination with history and clinical, spine and neurological examination.
   b. Confirm whether the patient is in the same or a different Class and Subclass in the GSCI Classification
   c. Review the intervention tables and suggest an alternative intervention for the Class and Subclass if available
   d. Refer to another level of care if no further intervention from the intervention tables is available in the clinical setting

4. **The symptoms have gotten worse since the last evaluation**
   a. Re-examination with more detailed history and examination.
   b. Confirm whether the patient is in the same or a different Class and Subclass in the GSCI Classification
   c. Review the intervention tables and suggest an alternative intervention for the Class and Subclass if available
   d. Refer to another level of care if no further intervention from the intervention tables is available in the clinical setting

5. **New spine-related symptoms have developed**
   a. Manage as a new spinal disorder per the initial clinician assessment guide

III. Clinician Guide - Treatment Interventions

Card 1. Clinical Decision Aid for Class 0

<table>
<tr><td colspan="2" align="center">Class 0
No or minimal spine-related symptoms
Person has concerns about developing symptoms or disability
No interference with function
Patient is requesting information on the prevention of spinal pathology, spinal pain, or spine-related disability</td></tr>
<tr><td colspan="2"><u>Assessments</u>
Brief history to identify concern or patient reported risk factors or concerns
Based upon patient characteristics, identify if there are any unrecognized risk factors
Clinical examination if indicated or requested
<u>Interventions</u>
Education about primary prevention for any risk factors (e.g., injury prevention, diet and physical activity advice for osteoporosis prevention)
Educate about condition, prognosis, reassurance, advice to remain active, and self-care instructions
Provide education on management of co-morbidities</td></tr>
<tr><td align="center">Class 0a
No evidence of risk factors for spine disorders or pain</td><td align="center">Class 0b
One or more risk factors for spine disorders or pain
Marked concern about future possible disability</td></tr>
<tr><td>No further recommendations once all concerns have been addressed</td><td><u>Assessments</u>
Assess for red flags
Assess for psychosocial flags
<u>Interventions</u>
Educate about risk factor or concern, reassurance, advice to remain active, and self-care instructions
Address any psychosocial flags</td></tr>
</table>

Card 2. Clinical Decision Aid for Class I

Class I
Mild Spinal Pain (2 to 4/10 numeric pain scale)
No or minimal interference with function or activities
No neurologic deficits
No serious or systemic pathology (no red flags)

Assessment

- History and clinical examination
- Assess for psychosocial flags
- Assess for risk factors and comorbidities
- Imaging studies such as x-rays and MRI are not recommended

Intervention

Education

- Educate about condition, prognosis, reassurance, advice to remain active, and self-care instructions
- Education about primary prevention for any identified risk factors (e.g., injury prevention, diet and physical activity advice for osteoporosis prevention)

Symptom alleviation

- Consider manual therapy (short-term)
- Consider acupuncture (short-term)
- Consider non-opioid analgesics/muscle relaxants (short-term)

Recommended Self-care

- Regular exercise
- Heat/cold (home use)

**Class Ia.**<br>**Acute/subacute pain**	**Class Ib.**<br>**Chronic or recurrent pain**
No further care recommendations unless the pain increases or becomes chronic	• Consider alternative symptomatic care options<br>• Consider supervised psychosocial therapy<br>• Consider supervised exercise<br>• Manage/track risk factors/comorbidities<br>• Psychosocial self-care if indicated

Card 3. Clinical Decision Aid for Class II

<table>
<tr><th colspan="2">Class II
Moderate spinal pain (5 to 7/10 numeric pain scale)
Interference with function or activities, but not incapacitating
No neurological deficits,
No serious or systemic pathology (no red flags)</th></tr>
<tr><td colspan="2"><u>Assessment</u>
• History and clinical examination
• Assess for psychosocial yellow flags
• Assess for risk factors and comorbidities
• Imagine studies such as x-rays and MRI are not recommended unless symptoms associated with significant trauma</td></tr>
<tr><td colspan="2"><u>Intervention</u>
<u>Education</u>
• Educate about condition, prognosis, reassurance, advice to remain active, and self-care instructions
• Education about primary prevention for any identified risk factors (e.g., injury prevention, diet and physical activity advice for osteoporosis prevention)
<u>Symptom alleviation</u>
• Consider manual therapy (short-term)
• Consider acupuncture (short-term)
• Consider non-opioid analgesics/muscle relaxants (short-term)
<u>Recommended Self-care</u>
• Regular exercise
• Heat/cold (home use)</td></tr>
<tr><th>Class IIa
Acute or subacute</th><th>Class IIb
Chronic or recurrent</th></tr>
<tr><td><u>Assessment</u>
• Consider opioid analgesics (short-term and only if other interventions are not controlling pain and if pain is significantly impacting function)
• No further care recommendations unless the pain increases or becomes chronic</td><td><u>Assessments</u>
Diagnostic imaging (if not done previously)

<u>Intervention</u>
• Avoid long term opioid analgesics
• Inter/multidisciplinary team care
• Mind-body therapies (e.g. Yoga, Tai-Chi)
• Psychosocial self-care</td></tr>
</table>

Card 4. Clinical Decision Aid for Class II

<table>
<tr><th colspan="2">Class II
Severe Spinal pain (8 to 10/10 numeric pain scale)
Interference with function or activities, but not incapacitating
No neurological deficits,
No serious or systemic pathology (no red flags)</th></tr>
<tr><td colspan="2"><u>Assessment</u>
• History and clinical examination
• Assess for psychosocial yellow flags
• Assess for risk factors and comorbidities
• Diagnostic imaging (if not done previously)</td></tr>
<tr><td colspan="2"><u>Intervention</u>
<u>Education</u>
• Educate about condition, prognosis, reassurance, advice to remain active, and self-care instructions
• Education about primary prevention for any identified risk factors (e.g., injury prevention, diet and physical activity advice for osteoporosis prevention)
<u>Symptom alleviation</u>
• Consider manual therapy (short-term)
• Consider acupuncture (short-term)
• Consider non-opioid analgesics/muscle relaxants (short-term)
• Consider opioid analgesics (short-term and only if other interventions are not controlling pain)
<u>Recommended Self-care</u>
• Regular exercise
• Heat/cold (home use)</td></tr>
<tr><td>Class IIc
Acute or subacute</td><td>Class IId
Chronic or recurrent</td></tr>
<tr><td><u>Assessment</u>

No further care recommendations unless the pain increases or becomes chronic</td><td><u>Assessments</u>
<u>Intervention</u>
• Inter/multidisciplinary team care
• Consider antidepressants if indicated
• Mind-body therapies (E.g. Yoga, Tai-chi)
• Psychosocial self-care
• Consider surgery (if unresponsive to other interventions and surgical lesion confirmed)</td></tr>
</table>

Card 5. Clinical Decision Aid for Class III

<table>
<tr><td colspan="3">Class III
Spine-related neurological symptoms or deficits causing interference with function or activities
With or without pain or disability</td></tr>
<tr><td colspan="3"><u>Assessment</u>
• History, clinical examination. Greater focus on neurologic symptoms and examination.
• Assess for psychosocial flags. Except for acute emergency (IIIb)
• Assess for risk factors and comorbidities. Except for acute emergency (IIIb)
• Diagnostic imaging (if not done previously)
• Laboratory testing (if signs of systemic disease)</td></tr>
<tr><td>Class IIIa
Minor and non-progressive
No red flags for serious or systemic disease</td><td>Class IIIb
Acute, major and progressive
No red flags for serious or systemic disease</td><td>Class IIIc
Chronic, stable
No red flags for serious or systemic disease</td></tr>
<tr><td>Assessment</td><td>Assessment</td><td>Assessment</td></tr>
<tr><td>Monitor for any progression of neurological deficits</td><td>• Emergency imaging (if appropriate and available)
• Emergency laboratory testing (if signs of systemic disease)</td><td>Monitor for any progression of neurological deficits</td></tr>
<tr><td>Interventions</td><td>Interventions</td><td>Interventions</td></tr>
<tr><td><u>Manage as Class 0, I or II depending on the severity of pain symptoms</u>

Consider elective decompression surgery (if deficits increase or no spontaneous recovery with time)</td><td><u>Emergency referral</u>
• Avoid manual therapy or exercise until stable
• Surgical decompression if indicated
• Specialty neurological or rheumatologic specialty referral if no surgical lesion found</td><td><u>Manage as Class 0, I or II depending on the severity of pain symptoms</u>

Specific interventions unlikely to change chronic stable neurological deficits</td></tr>
</table>

Card 6. Clinical Decision Aid for Class IV

<table>
<tr><th colspan="2">Class IV
Severe structural bony spinal deformity, fracture or instability
With or without interference with function or activities
With or without neurological deficits</th></tr>
<tr><td>Class IVa
Stable, no related symptoms
No serious trauma or deformity
No serious or systemic disease (no red flags)</td><td>Class IVb
Symptoms related to spine-related concern
Acute (e.g., fracture) or chronic (e.g., scoliosis or instability)
No serious or systemic disease (no red flags)</td></tr>
<tr><td>Assessments</td><td>Assessments</td></tr>
<tr><td>• History, clinical examination
• Assess for psychosocial flags
• Assess for risk factors and comorbidities
• Review available imaging (Additional imaging unlikely to be necessary)</td><td>• History, clinical examination
• Consider emergency imaging if acute trauma
• Assess for psychosocial flags if chronic
• Assess for risk factors and comorbidities if chronic</td></tr>
<tr><td>Intervention</td><td>Interventions</td></tr>
<tr><td>Manage as Class 0</td><td><u>Manage as Class 0, I or II depending on the severity of pain symptoms</u>
• Emergency bracing and stabilization in acute trauma
• Consider bracing for deformity
• Consider fusion or stabilization surgery, if fracture
• Consider vertebral augmentation surgery, if compression fracture
• Consider deformity surgery for scoliosis if indicated by surgical guidelines</td></tr>
</table>

Card 7. Clinical Decision Aid for Class V

<table>
<tr><td colspan="3">Class V
Spine related symptoms caused by serious systemic disease determined by red flags or clinical history or examination
(e.g. Infection, inflammatory joint disease, neoplastic disease, neurologic disease, psychological disease)</td></tr>
<tr><td colspan="3"><u>Assessment</u>
• History, clinical examination
• Diagnostic imaging to confirm non-spinal pathology
• Laboratory testing (for infection, metabolic or inflammatory disorders)
• Determine the specialist most likely to be able to manage the pathology
• Assess for risk factors and comorbidities</td></tr>
<tr><td>Class Va
Acute and severe symptoms and pathology</td><td>Class Vb
Slowly progressive symptoms and pathology</td><td>Class Vc
Referred pain from non-spinal pathology</td></tr>
<tr><td>• Emergency diagnostic imaging
• Emergency laboratory testing (for infection, metabolic or inflammatory disorders)
• Emergency referral to appropriate specialist</td><td>Immediate but not-emergent imaging, laboratory investigation and referral to appropriate specialist</td><td>Immediate or emergency referral to appropriate specialist dependent on severity of symptoms and pathology</td></tr>
</table>

Appendix 5. Description of the proposed GSCI spine care services in Cross Lake, northern Manitoba.

Primary spine care: Utilizing the GSCI model, trained registered nurses (RNs) and general physicians at the Cross Lake Nursing Station (accessible weekdays and on call in evenings and weekends) will apply the GSCI triage system to identify patients who may benefit from conservative management. The nurse in charge, has 20 years of experience in the overseeing 8 RNs/community nurses. Two to three general physicians provide medical care on weekdays and on call services for a wide range of conditions.

- Patients with spine concerns (Class 0) will receive education about primary prevention for any risk factors (e.g., injury prevention, physical activity advice for osteoporosis prevention); reassurance and education about specific spine conditions, prognosis, advice to remain active, and self-care instructions. In addition, they will be provided with education on management of related co-morbidities (e.g. diabetes, depression) through an interprofessional team.
- People presenting with Class Ia-b, Class IIa-d, Class IIIa and IIIc spine pain will be referred to a primary spine care clinician (Dr. J.W. and M.A.G. will deliver care at Pimicikamak nursing station 5 days per week) for a course of chiropractic care consisting of patient education, and advice on self-care and regular home exercise and heat/cold application, referral to mind-body therapies, and manual therapy (spinal mobilization manipulation, traction) and/or acupuncture.
- If appropriate, an onsite general physician may prescribe pharmaceutical therapy (e.g. non-opioid analgesics/muscle relaxants (short-term); and if indicated, opioid analgesics short-term and only if other interventions are not controlling pain. Class II

Secondary and tertiary spine care: Patients not responding to locally available evidence-based care, or who have red flags requiring further investigation or contraindicating conservative therapy (Class IIIb, Class IVb, Class Va,b,c) will be referred for a telehealth consult from the Pimicikamak nursing station with Advanced Practice Physiotherapists (APPTs) at the Winnipeg Spine Assessment Clinic working in collaboration with orthopedic spine surgeons (https://www.winnipegspineprogram.com/team).

- Acting as secondary care gatekeepers, APPTs may refer patients who need further evaluation (advanced imaging, other diagnostic procedures) to an orthopedic spine surgeon in the Winnipeg Spine Program for telehealth consultation, who in turn may request specialized medical consultation (e.g., rheumatology, neurology, multidisciplinary health care team) for suspected inflammatory spondyloarthropathies or neurological disorders.
- Patients with the most complex spinal disorders will be transferred to Health Sciences Centre Winnipeg by Stars Air Ambulance for advanced imaging and/or specialized medical care or possible surgery (Classes III-V).
- After a period of spinal rehabilitation, the patient will be transferred back to Pimicikamak by air. Clinicians (RNs, GPs) from the nursing station will ensure continuity of care with regular follow-up with specialists and rehabilitation providers.

Community Movement Program: A community-based programs will include education reassurance, exercise and home care delivered by the Health Department in collaboration with the Nursing Station through First Nations band governments, and movement program delivered bi-weekly by a staff member from the Health Department at the staff Band Hall or UCN building community center. Radio and YouTube video messaging will cover topics such as traumatic spine injury prevention, delivered by Health Department staff in collaboration with community leaders.

Appendix 6. Risk and Risk Mitigation Strategies

Risk Category and Description	Risk level	Proposed Risk Management	Responsibility assigned to:
Language and cultural barriers	Low-medium	Recruit staff from the local areas where possible who have understanding of the context and culture of the project areas; translate and culturally adapt information sheets and questionnaires	Local PI; research coordinator
Clinical:<br><br>Community residents identified as possibly having a serious spine pathology while conducting the Community survey (CS)	Low (<5% of spine symptoms due to serious underlying disease such as cancer, infection, fracture, inflammatory arthropathies)	Train research personnel who will administer the CS in village households to 1) triage people with spine symptoms, and 2) refer individuals to the local clinical team or to the nearest healthcare facility if urgent.	Research/clinical coordinator overseeing research personnel (research assistants; MSc students)
Community spine program not sustained by community leaders	Medium	Develop strong partnership with community leaders, provide progress reports twice/year; regularly seek input from leaders in to identify viable solutions	Local PI; research coordinator
Project management (planning, scheduling, estimating, or communication)	Low-medium	Develop and regularly update Gantt chart	Co-PIs, local PI; research coordinator
Organizational (project dependencies, logistics, resources, budget)	Low	Develop and regularly update Gantt chart; implement audit system (internal, external, operational, IT, supplier, and quality),	Co-PIs, local PI; research coordinator
Technical (technology being used, interfaces, performance, and data quality)	Medium	have paper-based information and questionnaires available; use university-based communication systems where available; standardized data collection and data entry, and train RAs and graduate students.	Local PI; research coordinator

Appendix 7. Knowledge Management and Dissemination Plan

Opportunities	Tools
1. Integrated KT: Activities in this category are those woven into the research process where stakeholders are engaged and integrated throughout the project. These knowledge-users were involved in the design of this project and are part of the team from the beginning. They will continue to be active members of the research team when it is time for interpreting study results and shaping the messages that should be communicated. Many of the researchers, knowledge-users and collaborators in this project are practicing clinicians and decision-makers in their institutions and will be able to help sustain the implementation of the MoC into practice at the community and health center levels.	• Regular virtual meetings (face-to-face when possible) to seek input, review documents, update team members, and plan next steps;<br>• Signed memorandum of understanding between sites and WSC<br>• Signed contracts between universities for fund transfer.
2. End-of-grant KT: To reach researchers, manuscripts will be submitted to open access peer-reviewed journals for publication, and national or international conferences will be targeted to present the results. In order to reach many stakeholders locally, team members will also present the results at local conferences or within their institutions. People with spine problems, their families, and community residents who will have expressed the desire to, will be reached by the publication of a printed or electronic bulletin informing them of the progression of our work and of the results as they become available. In addition, health centers such as regional hospitals generally have public sections on their website where they provide patients and families with valuable health information as well as research results useful to them. Finally, healthcare providers and community health workers will be targeted using presentations at professional meetings and distributing progress reports and printed educational material at the community health centers and nursing stations.	• Scientific (n=4) and professional (n=3) journal articles<br>• National conferences (n=3-6), international conferences (n=3)<br>• Bi-annual progress reports targeting decision makers, village leaders/influencers<br>• Annual progress reports to funders<br>• Printed or electronic bulletin (every 4 months) informing interested patients, family and community residents of the progression of our work<br>• Presentations to local clinicians and community health workers and printed educational material

References

1. Canadian Pain Task Force. Chronic Pain in Canada: Laying a Foundation for Action. Health Canada. June. 2019.

2. Briggs A, Jordan J, Jennings M, Speerin R, Chua J, Bragge P, Slater H. A Framework to Evaluate Musculoskeletal Models of Care. Cornwall, UK: Global Alliance for Musculoskeletal Health of the Bone and Joint Decade. Available at: https://www.aci.health.nsw.gov.au/__data/assets/pdf_file/0020/338141/Framework-to-Evaluate-Musculoskeletal-MoC.pdf (Accessed July 29 2021). 2016.

3. Briggs AM, Shiffman J, Shawar YR, Åkesson K, Ali N, Woolf AD. Global health policy in the 21st century: Challenges and opportunities to arrest the global disability burden from musculoskeletal health conditions. Best Pract Res Clin Rheumatol. 2020;34(5):101549. https://doi.org/.https://doi.org/10.1016/j.berh.2020.101549.

4. Gedde M, THET. Partnership for Global Health. The Tropical Health and Education Trust (THET): Available at: https://www.thet.org/principles-of-partnership/ (Accessed February 14 2024). 2009.

5. Pfadenhauer LM, Gerhardus A, Mozygemba K, Lysdahl KB, Booth A, Hofmann B, et al. Making sense of complexity in context and implementation: the Context and Implementation of Complex Interventions (CICI) framework. Implement Sci. 2017;12(1):21. https://doi.org/.10.1186/s13012-017-0552-5.

6. World Health O. WHO guideline for non-surgical management of chronic primary low back pain in adults in primary and community care settings. Geneva: World Health Organization 2023 [Available from: file:///C:/Users/bussiera/Downloads/9789240081789-eng%20(1).pdf.

7. Rowe AK, Rowe SY, Peters DH, Holloway KA, Chalker J, Ross-Degnan D. Effectiveness of strategies to improve health-care provider practices in low-income and middle-income countries: a systematic review. The Lancet Global Health. 2018;6(11):e1163-e75. https://doi.org/.10.1016/S2214-109X(18)30398-X.

8. Whitehorn A, Fu L, Porritt K, Lizarondo L, Stephenson M, Marin T, et al. Mapping clinical barriers and evidence-based implementation strategies in low-to-middle income countries (LMICs). Worldviews Evid Based Nurs. 2021;18(3):190-200. https://doi.org/.10.1111/wvn.12503.

9. Pantoja T, Opiyo N, Lewin S, Paulsen E, Ciapponi A, Wiysonge CS, et al. Implementation strategies for health systems in low-income countries: an overview of systematic reviews. The Cochrane database of systematic reviews. 2017;9(9):CD011086-CD. https://doi.org/.10.1002/14651858.CD011086.pub2.

10. Pantoja T, Grimshaw JM, Colomer N, Castañon C, Leniz Martelli J. Manually-generated reminders delivered on paper: effects on professional practice and patient outcomes. Cochrane Database Syst Rev. 2019;12(12):Cd001174. https://doi.org/.10.1002/14651858.CD001174.pub4.

11. Légaré F, Lee-Gosselin H, Borduas F, Monette C, Bilodeau A, Tanguay D, et al. Approaches to considering sex and gender in continuous professional development for health and social care professionals: An emerging paradigm. Med Teach. 2018;40(6):1-5. https://doi.org/.10.1080/0142159X.2018.1483579.

12. Chance-Larsen K, Backhouse MR, Collier R, Wright C, Gosling S, Harden B, et al. Developing a national musculoskeletal core capabilities framework for first point of contact practitioners. Rheumatol Adv Pract. 2019;3(2):rkz036-rkz. https://doi.org/.10.1093/rap/rkz036.

13. Nguyen T, Graham ID, Mrklas KJ, Bowen S, Cargo M, Estabrooks CA, et al. How does integrated knowledge translation (IKT) compare to other collaborative research

approaches to generating and translating knowledge? Learning from experts in the field. Health Res Policy Syst. 2020;18(1):35. https://doi.org/.10.1186/s12961-020-0539-6.
14. Gupta M, Marsden S, Oluka T, Sharma R, Lucas H. Lessons learned from implementing e-Learning for the education of health professionals in resource-constrained countries. Electronic J e-Learning. 2017;15(2):144-55.
15. Cieza A, Causey K, Kamenov K, Hanson SW, Chatterji S, Vos T. Global estimates of the need for rehabilitation based on the Global Burden of Disease study 2019: a systematic analysis for the Global Burden of Disease Study 2019. Lancet. 2021;396(10267):2006-17. https://doi.org/.10.1016/s0140-6736(20)32340-0.
16. Rowe AK, Rowe SY, Peters DH, Holloway KA, Ross-Degnan D. The effectiveness of training strategies to improve healthcare provider practices in low-income and middle-income countries. BMJ Glob Health. 2021;6(1). https://doi.org/.10.1136/bmjgh-2020-003229.
17. Kongsted A, Ris I, Kjaer P, Hartvigsen J. Self-management at the core of back pain care: 10 key points for clinicians. Braz J Phys Ther (In Press). 2021;https://doi.org/10.1016/j.bjpt.2021.05.002. https://doi.org/.https://doi.org/10.1016/j.bjpt.2021.05.002.
18. Schulman-Green D, Jaser SS, Park C, Whittemore R. A metasynthesis of factors affecting self-management of chronic illness. J Adv Nurs. 2016;72(7):1469-89. https://doi.org/.10.1111/jan.12902.
19. Barlow JH, Williams B, Wright CC. Patient education for people with arthritis in rural communities: the UK experience. Patient Educ Couns. 2001;44(3):205-14. https://doi.org/.10.1016/s0738-3991(00)00196-8.
20. Bonsignore C, Brolis E, Lonescu A, Karusinova V, Mitkova Z, Raps F, et al. Patient empowerment and centredness. European Health Parliament. 2015 Available at: https://www.healthparliament.eu/wp-content/uploads/2017/09/EHP-papers_Patients-empowerment.pdf (Accessed July 29 2021). 2015.
21. Currie SR, Wang J. Chronic back pain and major depression in the general Canadian population. Pain. 2004;107(1-2):54-60. https://doi.org/.10.1016/j.pain.2003.09.015.
22. Allegri M, Montella S, Salici F, Valente A, Marchesini M, Compagnone C, et al. Mechanisms of low back pain: a guide for diagnosis and therapy. F1000 Fac Rev 2016;5. https://doi.org/.10.12688/f1000research.8105.2.
23. Foster NE, Mullis R, Hill JC, Lewis M, Whitehurst DGT, Doyle C. Effect of stratified care for low back pain in family practice (IMPaCT Back): a prospective population-based sequential comparison. Ann Fam Med. 2014;12. https://doi.org/.10.1370/afm.1625.
24. Glasgow RE, Davis CL, Funnell MM, Beck A. Implementing practical interventions to support chronic illness self-management. Jt Comm J Qual Saf. 2003;29(11):563-74. https://doi.org/.10.1016/s1549-3741(03)29067-5.
25. Ghai B, Gudala K, Asrar MM, Chanana N, Kanukula R, Bansal D. Development, validation and evaluation of a novel self-instructional module in patients with chronic non-specific low back pain. Indian J Anaesth. 2020;64(4):299-305. https://doi.org/.10.4103/ija.IJA_779_19.
26. Petkovic J, Duench S, Trawin J, Dewidar O, Pardo Pardo J, Simeon R, et al. Behavioural interventions delivered through interactive social media for health behaviour change, health outcomes, and health equity in the adult population. Cochrane Database of Syst Rev. 2021;10.1002/14651858.CD012932.pub2(5). https://doi.org/.10.1002/14651858.CD012932.pub2.
27. Woolf A, P. K, Simon G, Rastrick S, Health Education England NEaSfH. Musculoskeletal core capabilities framework for first point of contact practitioners 2018.

Available at https://www.csp.org.uk/system/files/musculoskeletal_framework2.pdf (Accessed July 29 2021). 2018.
28. Haldeman S, Johnson CD, Chou R, Nordin M, Côté P, Hurwitz EL, et al. The Global Spine Care Initiative: classification system for spine-related concerns. European Spine Journal. 2018;27(6):889-900. https://doi.org/.10.1007/s00586-018-5724-8.
29. Haldeman S, Johnson CD, Chou R, Nordin M, Côté P, Hurwitz EL, et al. The Global Spine Care Initiative: care pathway for people with spine-related concerns. Eur Spine J. 2018;27(Suppl 6):901-14. https://doi.org/.10.1007/s00586-018-5721-y.
